# One species in eight: DNA barcodes from type specimens resolve a taxonomic quagmire

**DOI:** 10.1111/1755-0998.12361

**Published:** 2015-01-05

**Authors:** Marko Mutanen, Mari Kekkonen, Sean W. J. Prosser, Paul D. N. Hebert, Lauri Kaila

**Affiliations:** ^1^ Biodiversity UnitDepartment of BiologyUniversity of OuluP.O. Box 3000FI‐90014OuluFinland; ^2^ Zoology UnitFinnish Museum of Natural HistoryUniversity of HelsinkiP.O. Box 17FI‐00014HelsinkiFinland; ^3^ Biodiversity Institute of OntarioUniversity of GuelphGuelphONN1G 2W1Canada

**Keywords:** Automatic Barcode Gap Discovery, Barcode Index Number, *Elachista*, GMYC, species delimitation, species delineation

## Abstract

Each holotype specimen provides the only objective link to a particular Linnean binomen. Sequence information from them is increasingly valuable due to the growing usage of DNA barcodes in taxonomy. As type specimens are often old, it may only be possible to recover fragmentary sequence information from them. We tested the efficacy of short sequences from type specimens in the resolution of a challenging taxonomic puzzle: the *Elachista dispunctella* complex which includes 64 described species with minuscule morphological differences. We applied a multistep procedure to resolve the taxonomy of this species complex. First, we sequenced a large number of newly collected specimens and as many holotypes as possible. Second, we used all >400 bp examine species boundaries. We employed three unsupervised methods (BIN, ABGD, GMYC) with specified criteria on how to handle discordant results and examined diagnostic bases from each delineated putative species (operational taxonomic units, OTUs). Third, we evaluated the morphological characters of each OTU. Finally, we associated short barcodes from types with the delineated OTUs. In this step, we employed various supervised methods, including distance‐based, tree‐based and character‐based. We recovered 658 bp barcode sequences from 194 of 215 fresh specimens and recovered an average of 141 bp from 33 of 42 holotypes. We observed strong congruence among all methods and good correspondence with morphology. We demonstrate potential pitfalls with tree‐, distance‐ and character‐based approaches when associating sequences of varied length. Our results suggest that sequences as short as 56 bp can often provide valuable taxonomic information. The results support significant taxonomic oversplitting of species in the *Elachista dispunctella* complex.

## Introduction

Since its introduction 12 years ago (Hebert *et al*. 2003a,b), DNA barcoding has been widely applied by taxonomists as indicated by hundreds of published taxonomic studies utilizing DNA barcodes (Teletchea 2010). This work has shown that DNA barcoding is particularly useful in its main function, that is rapid identification of specimens. Indeed, many studies have shown that DNA barcodes allow the unambiguous identification of more than 90% of species and typically place the remainder to a very small number of closely related species (Barrett & Hebert 2005; Kerr *et al*. 2009; Lukhtanov *et al*. 2009; Dinca *et al*. 2011; Hausmann *et al*. 2011, 2013).

Besides specimen identification, DNA barcodes help to support several other taxonomic tasks. An increasing number of species owe their initial discovery to the observation of barcode divergences among specimens thought to represent a single species (e.g. Hebert *et al*. 2004; Segerer *et al*. 2010; Wilson *et al*. 2010; Huemer & Mutanen 2012; Mutanen *et al*. 2012a,b, 2013; Yang *et al*. 2012; Landry & Hebert 2013). In addition, barcodes have been used for delineating specimens into putative species (e.g. Puillandre *et al*. 2012b; Kekkonen & Hebert 2014). The benefits of using DNA barcodes for species delineation are clear especially when employed with novel species delineation methods (e.g. General Mixed Yule‐coalescent GMYC (Pons *et al*. 2006; Monaghan *et al*. 2009; Fujisawa & Barraclough 2013). As delineation methods generate a clearly defined result, the outcome is repeatable, largely objective and results are easily comparable between studies.

In addition to species identification and delineation, taxonomic work includes another crucially important task: naming. After the discovery of a new species, a formal taxonomic description is needed. This requires an evaluation of the status of prior names. Primary type specimens are central to this process as each taxonomic binomen is attached to them. As there may be many alleged synonyms, each with their own primary type specimen(s), it is often not straightforward to rule out the possibility that a putative new species already has a valid name (i.e. is it truly new). Therefore, the examination of type specimen(s) is necessary to check the possible existence of name(s) available for each newly discovered species. Particular difficulty in reaching a decision often arises when type specimens are in poor condition or when species show only small or uncertain morphological divergence. DNA barcodes provide a useful source of additional information for associating types with newly delineated species (Puillandre *et al*. 2011). Because type specimens tend to be old, only partial sequences are usually recovered, but even short segments of the barcode region often provide enough information to associate types to other specimens (Hajibabaei *et al*. 2006; Meusnier *et al*. 2008; Shokralla *et al*. 2011). However, short sequences can create complications for efforts to link types with delineated species.

Because the identification of specimens, the delineation of species and the naming of newly discovered species are different tasks, they require different tools and procedures (see also Collins & Cruickshank 2013). Methods suitable for identification are supervised because identifications must be based on an existing reference database, while species delineation has a more exploratory nature, meaning that unsupervised approaches are employed. When type barcodes are full‐length, their association with delineated species is straightforward. It can be conducted simultaneously to the delineation of species using only unsupervised methods (i.e. type barcodes are grouped in the same way as other sequences). However, as noted above, barcode sequences recovered from type specimens tend to be short due to DNA degradation, and these short sequences considerably complicate the association procedure because species delineation methods are very sensitive to variation in sequence length (see Material and methods). As a result, the inclusion of short barcodes requires a two‐stage procedure where species delineation is separated from the association of types with species.

Both supervised and unsupervised methods can be divided into three categories: distance‐ (or similarity)‐, tree‐ and character‐ (or diagnostic)‐based. Methods relying on genetic distances [e.g. blast (Altschul *et al*. 1997), BIN (Ratnasingham & Hebert 2013), ABGD (Puillandre *et al*. 2012a)] and trees (usually gene trees) (e.g. neighbour‐joining (Saitou & Nei 1987), GMYC (Pons *et al*. 2006; Monaghan *et al*. 2009; Fujisawa & Barraclough 2013)] are most often applied, but some character‐based tools are also available [e.g. caos (Sarkar *et al*. 2008), blog (Bertolazzi *et al*. 2009; Van Velzen *et al*. 2012; Weitschek *et al*. 2013)]. Generally, these methods deliver rather congruent results (Ratnasingham & Hebert 2013; Kekkonen 2014), but supervised character‐based methods perform especially well with recently diverged species (Van Velzen *et al*. 2012). However, as all methods have drawbacks, it is reasonable to employ several methods (Carstens *et al*. 2013).

Neither the use of DNA barcodes nor other single‐locus approaches are without problems (Dupuis *et al*. 2012). There are several biological processes that may result in a deep intraspecific mitochondrial DNA split (Hurst & Jiggins 2005; Rubinoff *et al*. 2006). Such splits, with no further evidence of several species, have been reported in several groups (Hausmann *et al*. 2011; Kvie *et al*. 2012; Kodandaramaiah *et al*. 2013). Among the most important mechanisms by which mtDNA may produce a misleading taxonomic signal are historical polymorphism, hybridization followed by mitochondrial introgression, prolonged isolation of populations without speciation and selective sweeps of haplotypes induced by *Wolbachia* (Funk & Omland 2003; Hurst & Jiggins 2005; Rubinoff *et al*. 2006). Due to these problems, taxonomic revisions should not be based solely on DNA barcodes or any other single genetic marker, but sequence clusters likely to represent different species should be evaluated with additional characters such as morphology or nuclear loci.

Highlighting the complexity of species delimitation, cases where poorly documented taxonomic work combined with weakly justified splitting of species has resulted in a taxonomic dead‐end where no specimen can be reliably identified are not rare (Mutanen 2005). The European *Elachista dispunctella*–*triseriatella* complex (Lepidoptera: Elachistidae), subsequently referred to as the *E. dispunctella* complex, appears to represent one such case. The members of this species group are largely restricted to Europe. All species with known life history are leaf miners in grasses (Poaceae). Species occupy xerothermic habitats at both low and high altitudes. Based on published information, many taxa have a very restricted distribution. This pattern, in combination with the stated high number of species, makes this group potentially important in the conservation of biodiversity, but an accurate taxonomy would be necessary. The *E. dispunctella* complex formerly comprised just six species. The first three, dating back to the 1800s, were based on records from England, Austria and Sardinia, while the other three were described during the 1900s, the most recent addition in 1974 from Italy (see Appendix S1, Supporting information). The *E. dispunctella* complex was subsequently intensively studied by Traugott‐Olsen (1985, 1988, 1992) who partitioned it into 64 species. This rise in the species count reflected a somewhat typological concept of species with no intraspecific variation in characters assumed. Rating the importance of characters without explicit justification, considering slight differences in wing venation as the primary basis for sorting the specimens into ‘sections of species’ led to a situation where otherwise identical‐looking specimens were placed into distinct sections, and subsequently, other characters were used to differentiate the species within each section, with no comparison among species in different sections. This procedure, presuming no intraspecific variation, led to the situation where over a third of the newly described species (27 in all) were based on a single specimen. For example, 11 species were reported from one hillside in Austria, all but one described as new by Traugott‐Olsen, while six other new species were found at a single site in the Sierra Nevada in Spain. The species from these two localities show no morphological differences apart from alleged minor differences in their wing venation. The use of this character is problematic because a study focused on *Elachista* showed that wing venation characters such as those used by Traugott‐Olsen (1988, 1992) display intraspecific and even intra‐individual (due to asymmetry) variation far exceeding the divergence used for the recognition of the sections of species by Traugott‐Olsen (1992) (Albrecht & Kaila 1997). The species in the venation‐based sections were based on apparent differences in genitalic morphology following the examination of a single or at best a few specimens, but many of the differences employed for species diagnosis by Traugott‐Olsen (1988, 1992) in *Elachista* appear to reflect distortions in shape due to poor dissection or involve differences in structures known to vary within species in related groups (Kaila 1999, 2009; Kaila *et al*. 2001). Because type specimens are often damaged or their shapes are distorted by poor dissections, subsequent efforts to resolve their status have failed. As the current taxonomy of the *E. dispunctella* complex still relies on characters of dubious or rejected value, so no specimen can be identified with certainty (e.g. Kaila 2007, 2011a,b, 2012).

**Figure 1:**
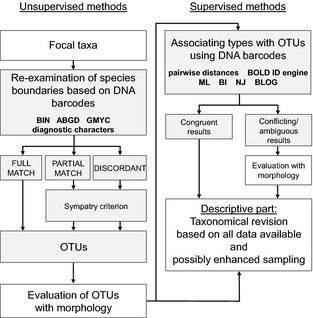
Schematic presentation of the study. The molecular data were divided into two subsets according to sequence length. Only barcode sequences >400 bp were employed for OTU delineation for re‐examination of species boundaries (unsupervised, exploratory methods), whereas both subsets were used for type association (supervised, reference‐based methods). Both molecular stages (grey boxes) were followed by evaluation based on morphological characters (male genitalia) to aid the elimination of possible errors caused by single‐locus DNA data. OTUs supported by both morphology and DNA will be preferred in the taxonomic revision unless evidence supporting conflicting boundaries (i.e. cryptic species) emerges during revisionary work.

Due to this situation, the *E. dispunctella* complex is in great need of taxonomic revision. However, many characteristics of *Elachista* make this work very challenging. These moths are tiny, possess few or no taxonomically useful external characters, and their genitalia structures are often difficult to interpret. As a consequence, a comprehensive taxonomic work would require a full‐time effort of many years. However, such slow process responds poorly to the needs created by the accelerating biodiversity loss, because obscurely defined species are difficult to protect. This study aims to resolve this dilemma through an integrated molecular and morphological approach (Fig. 1). The use of data from different sources is well accepted as the best practice for extremely challenging groups such as *Elachista* (Dayrat 2005). As a first step, we sequenced many newly collected specimens and as many holotypes as possible. Second, we used the >400 bp DNA barcodes to re‐examine species boundaries employing three unsupervised methods (BIN, ABGD, GMYC) with specified criteria how to handle discordant results, and examined diagnostic bases from each delineated putative species (here called operational taxonomic units, OTUs). Third, we studied the morphological characters of each OTU to avoid the limitations of single‐locus mtDNA. Finally, we associated short DNA barcodes from types with the delineated OTUs. In this step, we employed various supervised methods, representing all three categories (distance‐based: pairwise distances, and BOLD Identification System; tree‐based: neighbour‐joining, maximum likelihood and Bayesian inference; character‐based: blog).

## Material and methods

### Material acquisition and specimen sampling

Samples were obtained from the Finnish Museum of Natural History (University of Helsinki, Finland), National Museum of Natural History (Madrid, Spain), Naturhistorisches Museum (Wien, Austria), Netherlands Centre for Biodiversity Naturalis (Leiden, the Netherlands), The Natural History Museum (London, UK), Tiroler Landesmuseum Ferdinandeum (Innsbruck, Austria), Zoological Museum, Natural History Museum (Copenhagen, Denmark) and the private collections of Peter Buchner, Per Falck, Bob Heckford, Jari Junnilainen, Jari Kaitila, Kari and Timo Nupponen, Jukka Tabell, Zdenko Tokár and Christian Wieser.

Altogether 215 nontype specimens and 42 holotypes were sampled and sequenced. A single leg (usually a hind leg) was used for DNA extraction. Abdomens could not be utilized for DNA extraction because they had been permanently mounted following genital dissection. The nontype specimens were relatively fresh, the oldest collected in 1984, and 83% collected since 2000. We sequenced specimens from across the distribution of the *Elachista dispunctella* complex, directing particular effort to material collected at or near the type localities of the described species.

**Figure 2:**
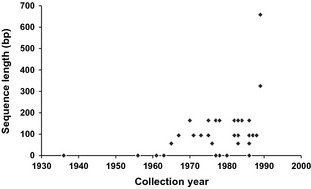
Length of DNA barcode sequences from 42 type specimens in the *Elachista dispunctella* complex.

We extracted DNA from 42 of the 64 holo‐ and lectotypes in the *E. dispunctella* complex (Table S2, Supporting information). One paratype (*E. svenssoni*) was also included, but it did not yield sequence data. With the exception of three species (*E. mannella*,* E. pocopunctella* and *E. svenssoni*) for which the year of collection of holotypes is unknown, all holotypes were collected during the 1900s, the youngest in 1989 and the oldest in 1936. The average age of the type specimens was 33 years (Fig. 2).

Collection data, Barcode Index Numbers (BINs) and GenBank Accession nos for the specimens are provided in Table S3 (Supporting information). All specimen and sequence data are available in the public dataset DS‐ELADIS in BOLD (www.boldsystems.org) (dataset DOI: http://dx.doi.org/10.5883/DS-ELADIS).

### Sequencing protocol for nontype specimens

All DNA sequencing was conducted at the Canadian Centre for DNA Barcoding (CCDB). Sequencing of nontype specimens followed routine DNA barcoding protocols described by Ivanova *et al*. (2006) and deWaard *et al*. (2008). In short, this involves silica membrane‐based DNA extraction followed by standard PCR using the primers listed on the sequence page for each record on BOLD although the LepF1‐LepR1 primer set was always used in the first trial. PCR was performed in a total reaction volume of 12.5 μL, and PCR products were assayed using the Invitrogen E‐gel 96 system. In cases where amplification was successful, 2 μL of the PCR product was sequenced. Sequencing followed protocols described in Hajibabaei *et al*. (2006) with each reaction performed in a final volume of 10.5 μL. Sequencing reactions were purified using either Sephadex columns or magnetic beads and sequenced on an ABI 3730 XL capillary sequencer.

### Sequencing protocol for type specimens

Due to the age of the type specimens (all >24 years), all molecular work with the exception of DNA sequencing was performed in a clean laboratory dedicated to the analysis of old specimens at the CCDB and employed sterile reagents and equipment. DNA extraction was performed in the same manner as nontype specimens except that the DNA was eluted from the silica membranes using 30 μL of molecular‐grade water vs. the standard elution volume of 40 μL. PCR was performed using various primers that target ~100‐ to 400 bp fragments of the COI barcode region. In cases where multiple overlapping fragments were recovered, they were combined to form a longer contiguous sequence. PCR cycling conditions were similar to those used for nontype specimens, but due to the low quantity of template DNA, 20 additional cycles were performed to amplify the target fragment to the concentration required for Sanger sequencing. Due to the increased risk of contamination, PCR products from type specimens were not visualized on E‐gels, but were directly sequenced using standard protocols. The resulting sequences were edited using codoncode v. 3.0.1 (CodonCode Corporation, Dedham, MA, USA). In addition, amino acids in sequences from type specimens were studied for nonsynonymous substitutions that are rare among closely related species and thus might reveal contamination events that would otherwise be overlooked.

### Sequence‐based OTU delineation

All sequences were aligned using the Aligner in bold v3.6 (Ratnasingham & Hebert 2007) and then inspected visually for stop codons and frameshift mutations in mega 5.0 (Tamura *et al*. 2011). DNA barcode sequences longer than 400 bp were used for OTU delineation with three methods: RESL algorithm which forms the basis of Barcode Index Number system (BIN; Ratnasingham & Hebert 2013), Automatic Barcode Gap Discovery (ABGD; Puillandre *et al*. 2012a) and General Mixed Yule‐coalescent (GMYC; Pons *et al*. 2006; Monaghan *et al*. 2009; Fujisawa & Barraclough 2013). The first two methods form OTUs based on different clustering algorithms and employ genetic distances, whereas GMYC is a model‐based likelihood method which seeks to determine the threshold between speciation and coalescent events from an ultrametric gene tree. The lineages crossing the threshold line represent the OTUs delineated by GMYC. No character‐based methods were used due to the lack of a feasible tool, but diagnostic base substitutions between delineated OTUs were examined.

Normally, sequences are automatically assigned to a BIN on the BOLD workbench v3.6, but these BIN assignments are based on the analysis of all barcode sequences on BOLD (currently 3.6 m). These BIN assignments are not strictly comparable with the other two delineation methods whose outcomes are based solely on analysis of the sequence collected in this study. To avoid this problem, we performed a RESL ‘stand‐alone’ analysis, using only the dataset in this publication (DS‐ELADIS). This version is not currently available for public use, but it will be released in the near future. ABGD analyses were performed at the web interface (http://wwwabi.snv.jussieu.fr/public/abgd/, web version April 11 2013) on 30 January 2014; for source code, see Appendix S4 (Supporting information) (downloaded on 30 January 2014) using a default value of relative gap width (*X* = 1.5) and all available distance metrics [p‐distance, JC69 (Jukes & Cantor 1969), K2P]. All other parameter values employed defaults. The General Mixed Yule‐coalescent (GMYC) method requires a fully‐resolved ultrametric gene tree as input for the analysis. We constructed a Bayesian inference tree in beast (Drummond *et al*. 2006; Drummond & Rambaut 2007) employing a coalescent tree prior (constant size; Kingman 1982). The coalescent prior was chosen due to the structure of the dataset (relatively small number of closely related species and generally high intraspecific sampling). However, previous results have indicated that tree prior has only minor effect on GMYC results (Kekkonen 2014). XML files were made with beauti v1.7.1 interface with the following settings: GTR+G+I substitution model, empirical base frequencies, four gamma categories, all codon positions partitioned with unlinked base frequencies and substitution rates. An uncorrelated relaxed lognormal clock model was used with rate estimated from the data and ucld.mean parameter with uniform prior with values 0 as a lower boundary and 10 as an upper boundary. All other settings were left as defaults. The applied XML file is available as Appendix S5 (Supporting information). The length of the MCMC chain was 40 000 000 sampling every 4000. beast runs were executed in Bioportal (Kumar *et al*. 2009) and CIPRES (Miller *et al*. 2010). The ESS values and trace files of runs were evaluated in tracer v1.5.0. Two independent runs were merged using logcombiner v1.7.1 with 20% burn‐in. A maximum clade credibility tree with a 0.5 posterior probability limit and node heights of target tree was constructed in treeannotator v1.7.1. Both single and multiple threshold model analyses were conducted in R (R Core Team 2012) using the ape (Paradis *et al*. 2004) and splits (Ezard *et al*. 2009) packages (for R code used for GMYC analyses, see Appendix S6, Supporting information). GMYC analyses were performed with haplotype data collapsed in alter (Glez‐Peña *et al*. 2010). alter is a web interface (http://sing.ei.uvigo.es/ALTER/), which was used to extract one representative of each haplotype (i.e. it discards all but one copy of each sequence in the dataset). GMYC should always be used with haplotype data as the inclusion of identical sequences can generate erroneous results (Monaghan *et al*. 2009).

The results of BIN, ABGD and GMYC were compared, and all OTUs were divided into three categories (FULL MATCH where all methods provide identical results, PARTIAL MATCH where two of three methods delineated the same OTU, DISCORDANT where all three methods show conflicting results) following the procedure introduced by Kekkonen & Hebert (2014). The PARTIAL MATCH and DISCORDANT categories involve cases where the results of the three delineation methods are in conflict, invoking a question as to which OTUs should be considered as valid and which should be rejected. According to the sympatry criterion (see Fig. 4, in Kekkonen & Hebert 2014), sympatric OTUs should be accepted while those in allopatry should be rejected. Although not a definitive argument, a sympatric split suggests the presence of separate species, while an allopatric split may simply reflect geographic variation in a single taxon.

Diagnostic bases of each OTU were studied on the BOLD workbench v3.6, employing the ‘Diagnostic Characters’ tool. Although this tool does not ordinarily allow the inclusion of singletons and doubletons, an exception was made with support from BOLD staff to allow the analysis of all OTUs. All codon positions were analysed. Due to the inclusion of singletons and doubletons, we recorded only pure diagnostic characters (*sensu* Sarkar *et al*. 2008).

### OTU evaluation with morphology

Due to the limitations of single‐locus mtDNA as the sole basis for taxonomic decisions, all OTUs delineated in the first phase were validated by examining the morphology of male genitalia (Fig. S7, Supporting information). We chose morphology instead of the examination of sequence diversity in one or more nuclear loci, because recovery of such data from small, often old specimens is difficult. The morphology of representative specimens from each OTU was examined using characters that have repeatedly been shown to match species boundaries in better known species groups of *Elachista* (see Mutanen *et al*. 2013 and references therein). The chief morphological differentiation in the *E*. *dispunctella* complex involves genitalia. The characters used were those detailed by Traugott‐Olsen & Nielsen (1977) and Kaila (2011c). Females could not be systematically studied, as they are unknown for several of the OTUs. In OTUs where both sexes were available, the possibility of using variation in female genitalia (Fig. S8, Supporting information) for their diagnosis was examined. The exploration of potential differences within and between OTUs was based on measurements of the relative sizes and shapes of genital structures. In addition to OTU validation, the male genitalia of all holotypes were also studied to enable comparison with conclusions based on DNA analysis.

### Association of type barcodes with OTUs

The inclusion of short sequences considerably altered the composition of OTUs in both the ABGD and GMYC analyses, indicating their inappropriateness for associating the short sequences from types with the longer sequences obtained from recently collected specimens. The RESL algorithm was not tested, but it likely suffers from the same problem as ABGD because both are distance‐based methods. As a consequence, we employed a different set of methods to associate the short barcode sequences from types with OTUs recognized in the OTU delineation phase. We employed three tree‐based approaches: maximum likelihood, Bayesian inference and neighbour‐joining; two distance based: comparison of pairwise distances and the matching algorithm of the BOLD Identification System; and one character based: blog (Bertolazzi *et al*. 2009; Van Velzen *et al*. 2012; Weitschek *et al*. 2013).

The neighbour‐joining analysis was performed with mega 5.0 using both K2P and uncorrected p‐distance models with pairwise deletion of missing data. The maximum‐likelihood analysis was performed with raxml blackbox (Stamatakis *et al*. 2008) using the GTR+G model and default bootstrap settings. Bayesian analysis was performed with the same data following the above‐mentioned protocol using beast, although no haplotype collapsing was conducted (for XML file, see Appendix S9, Supporting information).

**Figure 3:**
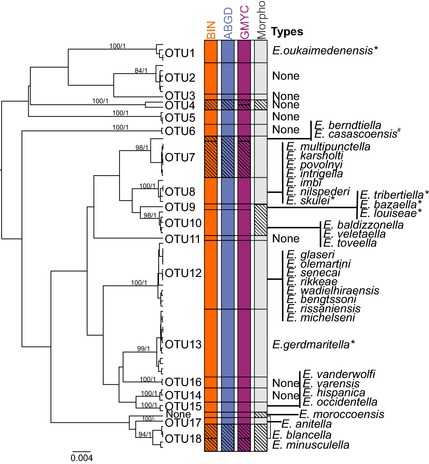
A Bayesian inference tree used for OTU delineation via GMYC (includes >400‐bp sequences) with bootstrap (from RAxML analysis) and posterior probability values (on the left). Coloured bars indicate delineated OTUs by different methods (Barcode Index Numbers BIN, Automatic Barcode Gap Discovery ABGD, General Mixed Yule‐coalescent GMYC and morphology) (in the middle). ‘Types’ includes all DNA barcodes of type specimens associated with OTUs in this study (on the right). Types marked with * have controversial results based on either conflicting results between the used methods or rather long distance to the nearest OTU (see text for further information). *E. casascoensis* (marked with #) is placed according to the distance measures, BLOG results and morphology as none of the tree‐based methods associated *E. casascoensis* with any OTU.

Problems may arise if a short sequence shows no difference from two longer ones which differ from each other by substitutions in regions outside the short sequence region. Under such conditions, the assignment of short sequences to a particular OTU can be misleading. To examine how neighbour‐joining and maximum likelihood behaved under these circumstances, we tested an arbitrary dataset of five slightly modified sequences from our data (nucleotides arbitrarily modified so that the short test fragment was identical with two long sequences of different OTUs) and observed that raxml (maximum likelihood) associated the short sequence with just one of the identical sequences, while mega and the ‘Taxon ID Tree’ tool in BOLD (neighbour‐joining) placed it as a distinct branch halfway between the two full‐length sequences that it matched. Both outcomes are unsatisfactory because they are potentially misleading as the real situation cannot necessarily be depicted in form of a single fully‐resolved tree. As we were not aware if any of the short sequences in our dataset were identical with long sequences of two or more species, we calculated pairwise K2P distances between each type and every one of the longer sequences to allow distance‐based assignments. Regarding treatment of missing data, the ‘pairwise comparison’ option of mega was used. Another distance‐based method used here, the BOLD Identification System, is a part of BOLD Systems interface. Each type barcode was searched against all barcode records with the minimum length of 500 bp on the BOLD database (analyses performed on January 2014). As only one barcode from a type specimen was full‐length (*E. moroccoensis*) and all other were shorter than 500 bp, the query sequences were not used as reference sequences (excluding *E. moroccoensis*) and thus matching a sequence with itself was not possible.

blog analyses were performed with blog v2.0 offline user interface. The method uses standard machine learning approach and requires two types of input: training and test datasets (Weitschek *et al*. 2013). blog creates logic classification formulas for each species (here OTUs) based on diagnostic characters and subsequently employs them to associate test sequences. We performed four analyses using different sequence lengths [654 bp (the original length), 162 bp, 93 bp, 54 bp]. The sequences were trimmed in mega. The rationale behind the length reduction was to maximize the number of shared characters (i.e. bases) between types and nontypes. After trimming, each dataset was divided into two parts. For training, we used all nontypes and *E. moroccoensis,* as in the OTU delineation phase, with all sequences named after their corresponding OTU. The test datasets included all barcodes from type specimens. The species names of types were changed to OTU names following the results of other type association methods (see Fig. 3). The change of names was made due to the requirements of blog as the names must match between the training and test datasets. Hence, blog was employed to test the results of other analyses. All parameters employed defaults.

## Results

### Sequencing success

Of the 215 nontype specimens, 194 (90.3%) yielded sequences and most (177) sequences were >600 bp. Sequencing success depended on specimen age, even though 16 of 21 unsuccessful specimens were collected after 2000 (logistic regression: estimate = −3.07, *n *=* *208, *P *=* *0.002). Failures in sequence recovery are unlikely to result from primer mismatching because those employed are effective across all lepidopteran families. Instead, sequencing failure likely reflects poor DNA preservation in some specimens. Sequencing success is generally known to decrease as a factor of specimen age (Meusnier *et al*. 2008), but exposure to certain chemicals leads to DNA degradation and the past preservation conditions of some specimens were not known.

About 75% (33 of 42) of the holotypes yielded some sequence information. There was no correlation between sequencing success or sequence length and specimen age (logistic regression: estimate = −1.82, *n *=* *37, *P *=* *0.069; Spearman rank correlation: *R*_S_ = −0.271, *n *=* *37, *P *=* *0.104, respectively). These sequences averaged 141 bp in length (Fig. 2), but a 658 bp barcode was recovered from the youngest type specimen, *E. moroccoensis* (collected 1989). However, 17 type specimens generated sequences <100 bp in length and only 56 bp were recovered from six specimens.

### OTU delineation

Altogether 191 DNA barcodes (>400 bp) were used for OTU delineation, including the full‐length sequence from the holotype of *E. moroccoensis* (78 haplotypes; the MSA used for OTU delineation is provided as a FASTA file in Appendix S10, Supporting information). The BIN analysis led to the recognition of 21 OTUs while ABGD resulted in a stable OTU count (19) with a range of prior intraspecific values (*P *=* *0.0077–0.0215) with both JC69 and K2P initial and recursive partitions (Fig. S11, Supporting information). As a result, this count (19) was used in the later analyses (Table 1). The recursive partitions also produced higher OTU counts (20‐62 OTUs) when the *P*‐value was 0.0046, but we adopted the OTU count of 19 due to its stability across a range of higher *P*‐values. Far more variable outcomes were produced when p‐distances were employed with OTU counts ranging from 1 to 35 (initial partition) and from 1 to 82 (recursive partitions) (Table 1). Because of their strong discordance from other results with ABGD and those obtained with other methods, these results are not considered further. Both single and multiple threshold models of GMYC outperformed the null model, indicating the presence of more than one species in the dataset (Table 2). The result from the single threshold model (22 OTUs) was adopted as the multiple threshold model (which resulted in 27 OTUs) did not improve fit to the data (*χ* ^*2*^ = 2.24, d.f. = 6, *P *<* *0.9).

**Table 1:** The number of OTUs recognized by Automatic Barcode Gap Discovery (ABGD) analyses among 191 COI sequences >400 bp using three distance metrics

Subst. model	X	Partition	Prior intraspecific divergence (P)								
			0.0599	0.0359	0.0215	0.0129	0.00774	0.00464	0.00278	0.00167	0.001
P	1.5	Initial	1	5	35	35	35	35	35	35	35
		Recursive	1	5	37	40	40	44	50	50	82
JC	1.5	Initial		0	19	19	19	19	19	19	19
		Recursive		0	19	19	19	20	25	27	62
K2P	1.5	Initial		0	19	19	19	19	19	19	19
		Recursive		0	19	19	19	20	25	27	62

X, relative gap width; P, p‐distance; JC69, Jukes‐Cantor; K2P, Kimura 2‐parameter.

**Table 2:** Results of the General Mixed Yule‐coalescent (GMYC) analyses for OTU formation (>400 bp sequences, *n* = 191, haplotypes = 78)

Analysis	Clusters (CI)	Entities (CI)	Likelihood_null_	Likelihood_GMYC_	Likelihood ratio	Threshold
Single	14 (13–15)	22 (20–25)	644.652	660.319	31.33367 (***)	−0.001613839
Multiple	16 (15–16)	27 (24–28)	644.652	661.441	33.57823 (***)	−0.001613839
						−0.001016101
						−0.0002361306

Clusters, OTUs delineated by GMYC with more than one specimen; Entities, all OTUs (clusters and singletons) delineated by GMYC; CI, confidence interval; Likelihood_null_: likelihood of the null model; Likelihood_GMYC_, likelihood of the GMYC model; Threshold, the threshold between speciation and coalescence processes; Single, single threshold model; Multiple, multiple threshold model. *** *P* < 0.001.

The three methods produced congruent results with three exceptions where OTUs were assigned to the PARTIAL MATCH category (see the diagonal lines on the BIN, ABGD and GMYC columns in Fig. 3). Both BIN and GMYC partitioned members of OTU7 and OTU18, whereas ABGD merged them into a single OTU. GMYC also divided OTU4, while BIN and ABGD did not. Following the sympatry criterion of Kekkonen & Hebert (2014), the split of OTU18 and OTU7 was ignored as it involved allopatric clusters. However, the two specimens of OTU4 were collected from the same place on the same day and thus distance‐dependent difference cannot explain the recognized genetic variation. Because of their sympatry, these two OTUs were kept separate at this stage. Following this rationale, the total number of OTUs was 19 among the nontype specimens. The total OTU count was 20 because the DNA barcode from the type specimen of *E. moroccoensis* did not cluster with any recently collected specimen (i.e. the type specimen formed its own OTU) (Fig. 3).

Diagnostic bases were designated based upon the analysis of the same 191 barcode sequences which were used for OTU delineation. Diagnostic characters were discovered for all OTUs, with from 2 to 14 diagnostic substitutions per OTU (Table 3).

**Table 3:** Diagnostic characters of delineated operational taxonomic units (OTUs)

OTU	Diagnostic characters
*Elachista* OTU1	11
*Elachista* OTU2	6
*Elachista* OTU3	7
*Elachista* OTU4	14
*Elachista* OTU5	7
*Elachista* OTU6	5
*Elachista* OTU7	7
*Elachista* OTU8	6
*Elachista* OTU9	4
*Elachista* OTU10	2
*Elachista* OTU11	2
*Elachista* OTU12	11
*Elachista* OTU13	9
*Elachista* OTU14	4
*Elachista* OTU15	4
*Elachista* OTU16	6
*Elachista* OTU17	4
*Elachista* OTU18	2
*Elachista* OTU19	3

### Evaluation of OTUs with morphology

The OTUs delineated on the basis of sequence variation in the DNA barcode region corresponded closely with morphological insights with three exceptions. First, specimens of the sister OTUs 9 and 10 appeared morphologically indistinguishable. Second, the holotype of *E. moroccoensis*, which formed an OTU of its own, and OTU18 appear indistinguishable by morphology. Third, the specimens belonging to OTU4, which was divided into two sympatric OTUs by GMYC showed no morphological differences. As the other OTUs were congruent between DNA and morphology, the presence of 17 OTUs was supported by this phase (Fig. 3). Table 4 provides a key for these OTUs based on their morphology.

**Table 4:** Morphological differentiation between operational taxonomic unit's (OTU's) in the *Elachista dispunctella* complex expressed as an identification key. Some species appear more than once in the key, as the characters sometimes display intraspecific variation, or variation in dissection technique and success may yield apparent although not real differences. For some species, the diagnostic morphological characters are found in one sex only

1. Forewing fringe scales distally grey forming grey distal line along termen__ 2
– Forewing fringe scales white, sometimes with single dark grey or brown tips of otherwise white scales __3
2. Juxta lobes with at least 5 distinctive setae; female bursa oval__ OTU 14
– Juxta lobes without or with at most two small setae; female bursa divided into two portions separated by median narrowing__OTU 13
3. Digitate process twice as long as juxta lobes__ OTU 18; *E. moroccoensis*
– Digitate process at most as long as juxta lobes__4
4. Phallus longer than valva__ OTU 15
– Phallus at most as long as valva__5
5. Uncus lobes narrow, three times as long as wide__OTU 5
– Uncus lobes at most twice as long as broad__6
6. Phallus with curved apex__7
– Phallus with straight apex__11
7. Forewing unicolorous white; digitate process laterally orientated__OTU 4
Forewing with scattered dark grey scales; digitate process posteriorly orientated__8
8. Length of phallus 5/6 of valva; juxta lobes as long as digitate process__OTU 7
– Length of phallus at most 2/3 of valva__9
9. Digitate process elongate, at least three times as long as wide__OTU 2
– Digitate process broad and triangular, length at most twice its width at base__10
10. Juxta lobes reduced__OTU 1
– Juxta lobes developed, as large as digitate process__OTU 3
11. Juxta lobes longer than uncus lobes__OTU 7
– Juxta lobes shorter than uncus lobes__12
12. Uncus lobes laterally produced, elongate, with pointed apex__OTU 6
– Uncus lobes posteriorly directed, with rounded or at most slightly lateroposteriorly conical apex__13
13. Phallus as long as valva__14
– Phallus shorter than valva__15
14. Female ostium bursae as wide as deep__ OTU 8
– Female ostium bursae three times as wide as deep__ OTU 9; OTU 10
15. Valva somewhat S‐shaped, narrowest medially; phallus basally significantly broader than distally__OTU 12
– Valva straight, parallel‐sided; phallus slender, hardly tapered toward apex__16
16. Uncus lobes as long as broad__OTU 17
– Uncus lobes longer than broad__17
17. Valva 3 x as long as its width basally__OTU 16
– Valva 4 x as long as its width basally__18
18. Valva 4 x as long as digitate process__OTU 11
– Valva 5 x as long as digitate process__OTU 9; OTU 10

### Associating type sequences with OTUs

Neighbour‐joining (Fig. S12, Supporting information), maximum likelihood (Fig. S13, Supporting information) and Bayesian inference (Fig. S14, Supporting information) analyses were performed with 223 sequences (the MSA used for type association is provided as a FASTA file in Appendix S15, Supporting information). Because none of these tree‐building methods is an actual species delineation method, they do not produce defined clusters so each type specimen was associated with the nearest OTU based on monophyly or sister group relationship. We considered sister lineages conspecific if their sequence divergence was less than 1%. All OTUs were monophyletic in all trees. In general, the three methods produced concordant associations of each type with a single OTU (Fig. 3, Table 5). Only one type, *E. casascoensis*, was inconsistently associated. It was placed as a sister lineage to OTUs 8 and 9 (NJ) or as a sister lineage to OTUs 9 and 10 (ML, BI). Aside from this discordance, there were two other cases with slight instability in type association with an OTU (*E. oukaimedenensis* and *E. gerdmaritella*). In both instances, the type barcode was nested within one OTU (*E. oukaimedenensis* with OTU1 and *E. gerdmaritella* with OTU13) in the maximum‐likelihood tree. However, Bayesian inference and neighbour‐joining analyses placed *E. oukaimedenensis* as a sister lineage to OTU1. *E. gerdmaritella* was included within OTU13 in the Bayesian tree, but was placed as a sister lineage to OTU16 in neighbour‐joining analysis. *E. skulei* was placed as a sister lineage to OTU8, and *E. tribertiella*,* E. bazaella* and *E. louiseae* came out as a sister lineage of OTU10 in all trees.

**Table 5:** The type specimens associated with OTUs according to tree‐based methods (maximum likelihood, Bayesian inference, neighbour‐joining with K2P) with bootstrap and posterior probability values. Discovered differences in amino acids between types and corresponding OTUs are also given. *E. casascoensis* and *E. moroccoensis* are excluded as they associated with none of the OTUs in tree‐based analyses

OTU	Types_ML_	Bootstrap_ML_	Types_BI_	PP	Types_NJ_	Bootstrap_NJ_	Amino acids
1	*E. oukaimedenensis*	88	*E. oukaimedenensis* a	0.97	*E. oukaimedenensis* a	63	
7	*E. berndtiella*	48	*E. berndtiella*	0.6	*E. berndtiella*	60	
8	*E*. *multipunctella*	79	*E. multipunctella*	0.99	*E. multipunctella*	30	
	*E*. *karsholti*		*E. karsholti*		*E. karsholti*		
	*E*. *povolnyi*		*E. povolnyi*		*E. povolnyi*		
	*E*. *intrigella*		*E. intrigella*		*E. intrigella*		
	*E*. *imbi*		*E. imbi*		*E. imbi*		
	*E*. *nilspederi*		*E. nilspederi*		*E. nilspederi*		
	*E. skulei* a		*E. skulei* a		*E. skulei* a		
9	*E. bazaella* a	69	*E. bazaella* a	1	*E. bazaella* a	53	
	*E. louiseae* a		*E. louiseae* a		*E. louiseae* a		
	*E. tribertiella* a		*E. tribertiella* a		*E. tribertiella* a		
10	*E. baldizzonella*	73	*E. baldizzonella*	0.99	*E. baldizzonella*	48	
	*E. veletaella*		*E. veletaella*		*E. veletaella*		
	*E. toveella*		*E. toveella*		*E. toveella*		
12	*E*. *glaseri*	56	*E. glaseri*	0.97	*E. glaseri*	32	
	*E*. *olemartini*		*E. olemartini*		*E. olemartini*		
	*E*. *senecai*		*E. senecai*		*E. senecai*		
	*E*. *rikkeae*		*E. rikkeae*		*E. rikkeae*		
	*E*. *wadielhiraensis*		*E. wadielhiraensis*		*E. wadielhiraensis*		
	*E*. *bengtssoni*		*E. bengtssoni*		*E. bengtssoni*		
	*E*. *rissaniensis*		*E. rissaniensis*		*E. rissaniensis*		
	*E*. *michelseni*		*E. michelseni*		*E. michelseni*		
13	*E. gerdmaritella*	84	*E. gerdmaritella*	0.98	NO		One difference
15	*E. vanderwolfi*	36	*E. vanderwolfi*	0.96	*E. vanderwolfi*	19	
	*E. varensis*		*E. varensis*		*E. varensis*		
	*E. hispanica*		*E. hispanica*		*E. hispanica*		
	*E. occidentella*		*E. occidentella*		*E. occidentella*		
16	NO		NO		*E. gerdmaritella* a	41	One difference
17	*E. anitella*	89	*E. anitella*	1	*E. anitella*	52	
	*E. blancella*	56	*E. blancella*	0.94	*E. blancella*	22	One difference
	*E. minusculella*	* *	*E. minusculella*	* *	*E. minusculella*	* *	* *

OTU, operational taxonomic unit; Types_ML_, Types_BI_, Types_NJ_, types associated with OTUs in maximum likelihood, Bayesian inference or neighbour‐joining (respectively) analyses; Bootstrap_ML_, Bootstrap_NJ_, boostrap values of OTUs including types in maximum likelihood or neighbour‐joining (respectively) analyses; PP, posterior probability values of OTUs including types in analysis.

a Type specimen associated as a sister to its corresponding OTU.

Comparison of pairwise distances largely supported results from the tree‐based methods, especially maximum likelihood and Bayesian inference with two exceptions (Table 6). First, *E. vanderwolfi* showed no divergence from OTU12 or OTU15. Second, *E. casascoensis* was associated with OTU7, but it remained distinct in all tree‐based results. Closer analysis indicated that the first case was an artefact caused by the limited overlap between type sequences and incomplete sequences from some recent specimens. All other sequences produced a result identical with the tree‐based type association. Barcode sequences from type specimens were also searched against records on BOLD. Based on the results of the BOLD Identification System, 25 of 31 types were matched with specimens on the database with more than 97.83% similarity (Table 7). The other six barcodes from type specimens were too short (56 bp) to gain a result. The outcome supported the result of pairwise distances as *E. casascoensis* was associated with OTU7. Otherwise, the results were congruent with other methods.

**Table 6:** Best correspondence (by least K2P distance) between the short sequences from 33 type specimens in the *Elachista dispunctella* complex and operational taxonomic units (OTUs). The second column indicates the length of the sequence in base pairs. The values in the columns 3–5 are minimum K2P distances between the type specimens and OTUs in question

Type	Seq. length	Best hit	2nd best hit	3rd best hit
*E. anitella*	164	OTU17 (0.000)	OTU18 (0.012)	OTU7 (0.031)
*E. baldizzonella*	164	OTU10 (0.000)	OTU9 (0.019)	OTU7 (0.019)
*E. bazaella*	94	OTU9 (0.011)	OTU7 (0.033)	OTU8, OTU10, OTU17, OTU18 (0.044)
*E. bengtssoni*	325	OTU12 (0.000)	OTU16 (0.038)	OTU1 (0.042)
*E. berndtiella*	164	OTU7 (0.000)	OTU9, OTU10, OTU13, OTU15 (0.025)	OTU18 (0.031)
*E. blancella*	164	OTU18 (0.006)	OTU17 (0.019)	OTU7 (0.038)
*E. casascoensis*	164	OTU7 (0.006)	OTU10 (0.012)	OTU9 (0.012)
*E. gerdmaritella*	94	OTU13 (0.011)	OTU16 (0.022)	OTU7 (0.044)
*E. glaseri*	94	OTU12 (0.000)	OTU15 (0.022)	OTU7 (0.027)
*E. hispanica*	94	OTU15 (0.000)	OTU12 (0.013)	OTU7 (0.022)
*E. imbi*	164	OTU8 (0.000)	OTU9 (0.025)	OTU10 (0.031)
*E. intrigella*	164	OTU8 (0.000)	OTU9 (0.025)	OTU10 (0.031)
*E. karsholti*	94	OTU8 (0.000)	OTU9 (0.033)	OTU5, OTU7, OTU10 (0.044)
*E. louiseae*	56	OTU9 (0.018)	OTU7 (0.037)	OTU6, OTU8, OTU10, OTU17, OTU18 (0.056)
*E. michelseni*	56	OTU12 (0.000)	OTU15 (0.018)	OTU1, OTU16 (0.037)
*E. minusculella*	56	OTU18 (0.000)	OTU17 (0.018)	OTU5 (0.037)
*E. moroccoensis*	658	N/A	OTU18 (0.045)	OTU17 (0.051)
*E. multipunctella*	164	OTU8 (0.006)	OTU9 (0.031)	OTU10 (0.038)
*E. nielspederi*	164	OTU8 (0.000)	OTU9 (0.025)	OTU10 (0.031)
*E. occidentella*	164	OTU15 (0.000)	OTU7 (0.025)	OTU12 (0.028)
*E. olemartini*	94	OTU12 (0.000)	OTU15 (0.022)	OTU7 (0.027)
*E. oukaimedenensis*	94	OTU1 (0.014)	OTU7 (0.044)	OTU12, OTU15, OTU17 (0.055)
*E. povolnyi*	56	OTU8 (0.000)	OTU5, OTU7, OTU9, OTU10 (0.037)	OTU18 (0.057)
*E. rikkeae*	94	OTU12 (0.000)	OTU15 (0.022)	OTU7 (0.027)
*E. rissaniensis*	164	OTU12 (0.000)	OTU7 (0.031)	OTU10, OTU13, OTU15, OTU16 (0.038)
*E. senecai*	93	OTU12 (0.000)	OTU15 (0.022)	OTU7 (0.027)
*E. skulei*	164	OTU8 (0.019)	OTU7 (0.031)	OTU9, OTU10, OTU15, OTU17, OTU18 (0.038)
*E. toveella*	164	OTU10 (0.000)	OTU7, OTU9 (0.019)	OTU8, OTU12 (0.031)
*E. tribertiella*	90	OTU9 (0.011)	OTU7 (0.034)	OTU8, OTU10, OTU17, OTU18 (0.046)
*E. wadielhiraensis*	164	OTU12 (0.000)	OTU7 (0.025)	OTU10, OTU13, OTU15, OTU16 (0.031)
*E. vanderwolfi*	56	OTU12, OTU15 (0.000)	OTU1 (0.018)	OTU5 (0.037)
*E. varensis*	94	OTU15 (0.000)	OTU12 (0.013)	OTU7 (0.022)
*E. veletaella*	56	OTU10 (0.000)	OTU7 (0.027)	OTU8, OTU9 (0.037)

**Table 7:** Results of BOLD ID engine. Type sequences were searched against all barcode records on BOLD with a minimum sequence length of 500 bp

Types	BOLD ID engine (# matching sequences)	Result with highest similarity (%)
*Elachista oukaimedenensis*	OTU1 (3)	97.85
*E. berndtiella*	OTU7 (7)	100
*E. casascoensis*	OTU7 (2)	99.38
*E. multipunctella*	OTU8 (7)	99.38
*E. karsholti*	OTU8 (16)	100
*E. povolnyi*		N/A
*E. intrigella*	OTU8 (7)	100
*E. imbi*	OTU8 (7)	100
*E. nilspederi*	OTU8 (7)	100
*E. skulei*	OTU8 (7)	98.15
*E. bazaella*	OTU9	98.92
*E. louiseae*		N/A
*E. tribertiella*	OTU9	98.89
*E. baldizzonella*	OTU10 (7)	100
*E. veletaella*		N/A
*E. toveella*	OTU10 (7)	100
*E. glaseri*	OTU12 (20)	100
*E. olemartini*	OTU12 (20)	100
*E. senecai*	OTU12 (20)	100
*E. rikkeae*	OTU12 (20)	100
*E. wadielhiraensis*	OTU12 (1)	100
*E. bengtssoni*	OTU12 (20)	100
*E. rissaniensis*	OTU12 (20)	100
*E. michelseni*		N/A
*E. gerdmaritella*	OTU13 (20)	98.92
*E. vanderwolfi*		N/A
*E. varensis*	OTU15 (9)	100
*E. hispanica*	OTU15 (9)	100
*E. occidentella*	OTU15 (9)	100
*E. anitella*	OTU17 (2)	100
*E. blancella*	OTU18 (7)	99.38
*E. minusculella*		N/A
*E. moroccoensis*	*E. moroccoensis* a (1)	100

OTU, operational taxonomic unit.

a Type specimen associated with itself.

In the results of the blog analyses, the shortest sequence length (54 bp) produced the highest number of associated types as all but two were correctly linked with an OTU (i.e. they supported the results of the other association methods) (Table 8). The two discordant results included the type of *E. skulei*, which was associated with OTU7 instead of OTU8, and *E. minusculella*, which was not associated with any OTU. By contrast, the blog analysis with full‐length sequences (654 bp) resulted in only three types being correctly associated with OTU9. All the rest were unlinked to any OTU. The full‐length barcode of *E. moroccoensis* was only associated with itself. The logic formulas of blog included one to four distinctive characters for each OTU and changed with the sequence length (Appendix S16, Supporting information).

**Table 8:** Results of four blog analyses (654, 162, 93, 54 bp). The type of *E. moroccoensis* is excluded from this table as it only associated with itself in all analyses

	Sequence length (bp)															
	654				162				93				54			
	# corr.	% corr.	# wrong	# n.c.	# corr.	% corr.	# wrong	# n.c.	# corr.	% corr.	# wrong	# n.c.	# corr.	% corr.	# wrong	# n.c.
OTU1	0	0	0	1	1	100	0	0	1	100	0	0	1	100	0	0
OTU2	0	0	0	0	0	0	0	0	0	0	0	0	0	0	0	0
OTU3	0	0	0	0	0	0	0	0	0	0	0	0	0	0	0	0
OTU4	0	0	0	0	0	0	0	0	0	0	0	0	0	0	0	0
OTU5	0	0	0	0	0	0	0	0	0	0	0	0	0	0	0	0
OTU6	0	0	0	0	0	0	0	0	0	0	0	0	0	0	0	0
OTU7	0	0	0	2	2	100	0	0	2	100	0	0	2	100	0	0
OTU8	0	0	0	7	5	71.43	1	1	5	71.43	1	1	6	85.71	1	0
OTU9	3	100	0	0	3	100	0	0	3	100	0	0	3	100	0	0
OTU10	0	0	0	3	3	100	0	0	3	100	0	0	3	100	0	0
OTU11	0	0	0	0	0	0	0	0	0	0	0	0	0	0	0	0
OTU12	0	0	0	8	8	100	0	0	8	100	0	0	8	100	0	0
OTU13	0	0	0	1	1	100	0	0	1	100	0	0	1	100	0	0
OTU14	0	0	0	0	0	0	0	0	0	0	0	0	0	0	0	0
OTU15	0	0	0	4	1	25	0	3	3	75	0	1	4	100	0	0
OTU16	0	0	0	0	0	0	0	0	0	0	0	0	0	0	0	0
OTU17	0	0	0	1	1	100	0	0	1	100	0	0	1	100	0	0
OTU18	0	0	0	2	1	50	0	1	1	50	0	1	1	50	0	1

# corr., number of correctly classified elements; % corr., percentage of correctly classified elements; # wrong, number of wrongly classified elements; # n.c., number of unclassified elements; OTU, operational taxonomic unit.

In general, the 658 bp sequence from the type of *E. moroccoensis* formed a unique OTU in all analyses, supporting its distinctiveness from the other specimens (Fig. 3). The type of *E. casascoensis* did not cluster with any OTU in the tree‐based analyses, but both distance and character analyses associated both it and *E. berndtiella* with OTU7 (Table 9). All other type specimens were also associated with OTUs. In fact, eight holotypes were grouped within OTU12, while six types were associated with OTU8 with one additional holotype (*E. skulei*) as a close sister lineage. The results of the blog analyses produced discordant outcomes as they associated *E. skulei* with OTU7. The barcode results associated two sets of three to four holotypes with OTU10 and OTU15. Three other types (*E. tribertiella, E. bazaella* and *E. louiseae*) possessed identical barcode sequences and formed the sister lineage for OTU9. Two other holotypes were associated with an OTU (*E. oukaimedenensis* with OTU1; *E. gerdmaritella* with OTU13) as discussed earlier.

**Table 9:** Type association based on DNA barcodes. Numbers in cells correspond to the operational taxonomic unit (OTU) where each method linked a given type specimen. Discordant results are marked in bold

Types	NJ	ML	BI	Pw. dist.	BOLD ID	blog
*Elachista oukaimedenensis*	1^a^	1	1^a^	1	1	1
*E. berndtiella*	7	7	7	7	7	7
***E. casascoensis***	**8,9**	**9,10**	**9,10**	7	7	7
*E. multipunctella*	8	8	8	8	8	8
*E. karsholti*	8	8	8	8	8	8
*E. povolnyi*	8	8	8	8	N/A	8
*E. intrigella*	8	8	8	8	8	8
*E. imbi*	8	8	8	8	8	8
*E. nilspederi*	8	8	8	8	8	8
***E. skulei***	8^a^	8^a^	8^a^	8	8	**7**
*E. bazaella*	9^a^	9^a^	9^a^	9	9	9
*E. louiseae*	9^a^	9^a^	9^a^	9	N/A	9
*E. tribertiella*	9^a^	9^a^	9^a^	9	9	9
*E. baldizzonella*	10	10	10	10	10	10
*E. veletaella*	10	10	10	10	N/A	10
*E. toveella*	10	10	10	10	10	10
*E. glaseri*	12	12	12	12	12	12
*E. olemartini*	12	12	12	12	12	12
*E. senecai*	12	12	12	12	12	12
*E. rikkeae*	12	12	12	12	12	12
*E. wadielhiraensis*	12	12	12	12	12	12
*E. bengtssoni*	12	12	12	12	12	12
*E. rissaniensis*	12	12	12	12	12	12
*E. michelseni*	12	12	12	12	N/A	12
***E. gerdmaritella***	**16** a	13	13	13	13	13
***E. vanderwolfi***	15	15	15	**12,15**	N/A	15
*E. varensis*	15	15	15	15	15	15
*E. hispanica*	15	15	15	15	15	15
*E. occidentella*	15	15	15	15	15	15
*E. anitella*	17	17	17	17	17	17
*E. blancella*	18	18	18	18	18	18
*E. minusculella*	18	18	18	18	N/A	N/A
*E. moroccoensis*	None	None	None	N/A	N/A	N/A

NJ, Neighbour‐Joining; ML, maximum likelihood; BI, Bayesian inference; Pw. dist., pairwise distances; BOLD ID, BOLD Identification system; blog, blog analysis with the sequence length of 54 bp.

a Placed as a sister lineage.

### Correspondence of type morphology with OTUs

The holotypes of *E. moroccoensis*,* E. blancella*,* E. minusculella* and the recent specimens of OTU18 appear indistinguishable by morphology, but as *E. moroccoensis* had a distinctive barcode, it may represent a cryptic species. Morphology supports the association of the *E. casascoensis* holotype with OTU7 that also includes the holotype of *E. berndtiella*, with no character found to differentiate the recognition of *E. casascoensis* from the widespread OTU7. The type of *E. gerdmaritella*, which was differently placed in the tree‐based methods, shows no morphological differences from specimens in OTU13. The OTUs with several matching holotypes (e.g. 8, 10, 12, 15) each include specimens that are indistinguishable morphologically. The same holds true with the holotypes associated with these OTUs as they also do not differ from each other in any other way except on the basis of the wing venation traits whose value as species‐diagnostic trait is questionable (Albrecht & Kaila 1997). Hence, the results from morphology correspond closely with those from barcodes.

## Discussion

This study was initially motivated by the uncertain taxonomy of the *Elachista dispunctella* complex which likely derives from two factors. First, many *Elachista* species show subtle morphological diversification (Kaila 2011c; Mutanen *et al*. 2013). Second, prior taxonomic work has sometimes created an apparent proliferation of names (cf. Albrecht & Kaila 1997). Our study asked whether DNA barcodes from type specimens could help to clarify the true of species boundaries in the *E. dispunctella* complex. To accomplish this, we employed a procedure which combined various unsupervised and supervised methods together with the inspection of morphological characters. This approach was necessary due to the short length of the DNA barcodes of the type specimens as the unsupervised analysis of all sequences was not possible. Following this procedure, 20 OTUs were delineated based on DNA barcodes (including the OTU of *E. moroccoensis*), and 17 of these OTUs were still recognized after morphological study. These 17 OTUs will be prioritized for recognition as species in a future taxonomic revision unless evidence supporting the distinctiveness of additional OTUs emerges during the revisionary work. The status of other apparently cryptic species should also be examined with further molecular methods.

These moths are small (10 mm wingspan), and the abdomens of all types are permanently mounted so they are unavailable for DNA extraction. As a result, little tissue material from types was available for analysis, but we recovered partial barcode sequences from the holotypes of 33 species, making it possible to see whether their sequences showed congruence with one or more of the OTUs delineated in the study of recent specimens. Because the number of OTUs recovered from modern specimens was less than the number of type specimens analysed, one would expect many sequences from type specimens not to find a match if each described species is distinct. In fact, there was only a single case of such divergence, the holotype of *E. moroccoensis* represented a unique OTU, with 4.5% divergence from the closest OTU. As a consequence, the validity of *E. moroccoensis* as separate species is supported by its sequence divergence. By contrast, there were numerous cases where the barcode sequence from several type specimens matched a particular OTU with the most extreme case involving eight type specimens associated with OTU12 (cf. Fig. 3). This result suggests that prior taxonomic work has led to considerable oversplitting, a conclusion fortified by the close agreement between morphological results and those from barcode analysis (cf. Table 4). We conclude that the sequences recovered, even the shortest ones, are a valuable enabler for a comprehensive integrative taxonomic revision of the group. Because such a revision will require the reassessment of all 64 nominal species, it is beyond the scope of the present investigation.

### Evaluation of the procedure employed

Due to the short length of the DNA barcodes recovered from type specimens, we conducted an initial phase of OTU delineation prior to associating types to these OTUs. This approach served two goals: re‐examining species belonging to the *E. dispunctella* complex and providing clearly defined groups to be used in the type association phase. In the study of Puillandre *et al*. (2011), type barcodes were associated employing tree‐based methods (NJ and BI) without a separate delineation phase. This simplifies the procedure, but most of the benefits derived from DNA‐based methods for species delineation methods (e.g. accuracy, objectivity and repeatability) are not utilized. As methods and criteria used in the delineation phase were detailed in a previous study (Kekkonen & Hebert 2014), further discussion on this approach would not offer any new aspects and is thus excluded.

The majority (15 of 19) of the putative species (OTUs) delineated through barcode analysis were congruent with morphological characters, and all four cases of discordance involved cases of splits recognized by DNA barcodes where the groups showed no morphological divergence (Fig. 3). The few cases of discordance indicate that most of our data were free from the potential problems caused by single‐locus DNA. The most dubious result of barcode‐based delineation was the division of OTU4, a result that was likely an artefact of small sample sizes. The same factor may explain the discordant result for OTUs 9 and 10, as all three sequence‐based delineation methods recognized OTU9. The most convincing evidence for cryptic species was offered by *E. moroccoensis* as its full‐length barcode was always separated from the other sequences.

The tree‐ and distance‐based methods employed for type association generally produced results concordant with those from morphology. The results were also mostly congruent between different DNA‐based methods, indicating that tree‐ and distance‐based methods can be employed even with very short sequences, such as those often recovered from type specimens. However, our results revealed some drawbacks in analysing short sequences with certain methods. Tree‐based methods, such as neighbour‐joining and maximum likelihood, have the pitfall that they can place a short sequence on a distinct node even if it is identical with several different full‐length sequences. This problem can be averted by determining whether each holotype is equidistant from several full‐length barcodes or just one. This issue was detected for the identical sequences of *E. blancella* and *E. minusculella* which were differently placed, although they were still associated with the same OTU (OTU18) (Figs S12 and S13, Supporting information). Bayesian inference analysis avoided this error as it placed the two type specimens in the same cluster (Fig. S14, Supporting information). Also, the placement of *E. casascoensis*, which was associated with OTU7 by distance‐ and character‐based methods and morphology, was rather unstable in tree‐based analyses. Comparison of pairwise distances can yield a biased outcome when overlap between the target sequence and reference sequences is partial. Therefore, in critical cases, such reference sequences should be excluded from analysis. This problem was detected in one case (*E. vanderwolfi*).

blog appeared to be particularly sensitive to variation in sequence length because the analysis of the dataset with both long and short sequences resulted in only three correctly assigned types. By contrast, when the length of all sequences was trimmed to 162 bp, the number of correctly assigned types rose to 26 with the shortest sequences (54 bp) producing the highest number of correct matches. This indicates that blog should only be used with DNA sequences of equal or very similar length. In addition, it was the only method (out of six) that delivered an unambiguous result (i.e. type barcode is either associated with a particular OTU or not), while the results from other methods are more subjective. This feature makes blog, or any other supervised method based on diagnostic characters, recommended provided that the sequences are either similar in length or are trimmed to meet this requirement.

Because of the increasing efforts being directed towards the barcoding of type specimens, often yielding only short sequences, the potential pitfalls of both tree‐building methods and distance matrices should be considered. The tendency of neighbour‐joining analysis to generate fully‐resolved trees (up to the level of entirely identical sequences) even when this is not supported by the data is an obvious problem.

### Future of type barcoding

Recent progress in the recovery of DNA sequences provides hope that nondestructive sampling (e.g. Hunter *et al*. 2008) of type specimens will soon become a routine part of establishing a stable nomenclature, especially in groups that are currently intractable because of the poor condition of type specimens or the close morphological similarity of species. Our results support earlier conclusions that even short segments of the barcode region usually provide enough information to associate type material with other specimens (Meusnier *et al*. 2008; Lees *et al*. 2010; Shokralla *et al*. 2011). In addition, different methods are available to acquire the whole barcode. For instance, primer walking can recover the entire region through sequencing several overlapping fragments, an approach already used for moths (Hausmann *et al*. 2009a,b; Wilson *et al*. 2010; Rougerie *et al*. 2012), and fruit flies (van Houdt *et al*. 2010). For example, Strutzenberger *et al*. (2012) recovered complete barcodes from 95% of the abdomens that they sampled from 79‐ to 157‐year‐old *Eois* moths. Unfortunately, because all holotypes examined in this study lacked an abdomen, it was necessary to analyse just a single leg, providing much lower concentrations of DNA.

Techniques to recover DNA barcodes from old specimens are developing rapidly. It is likely that both Sanger‐based and next‐generation sequencing techniques will soon recover DNA sequences from older and more degraded specimens than is routine today. This prospect opens new opportunities for resolving taxonomic problems that require a better understanding of the identity of the name‐bearing specimen. In taxonomy, type specimens are central in associating taxa with names. As shown in this study, DNA barcoding can help to establish this linkage in a powerful way. Because DNA extraction can now be performed in a nondestructive manner, there is no basis for opposition to the analysis of type specimens. We find it inconsistent that the policies of some museums do not permit tissue sampling from type material for DNA analysis, although other far more destructive investigations (e.g. genital dissection) are permitted.

Species delimitation is inherently subjective, reflecting the variety of species concepts of species and the difficulties associated with deciding the status of allopatric populations. Such situations are problematic in data management, maintaining checklists and keeping track of new information of species distributions, in a wide variety of databases of importance for numerous applications. In less‐studied groups of organisms, new samples may change the prevailing taxonomic perspective, for instance by making it possible to better quantify intra‐ or interspecific variation which may shift conclusions on species boundaries. Sometimes, poorly justified approaches to distinguish taxa can cause overlumping or oversplitting of taxa. Both of these phenomena cause problems: lumping obscures true diversity and splitting overestimates it. Both lumping and splitting hamper the accurate identification of specimens biasing the interpretation of variation in diagnostic features. In the species complex considered in this study, oversplitting has led to the situation where no specimen in this group of European moths, generally assumed to be well known, could be correctly identified since 1992. A reliable morphology‐based taxonomic revision has been extremely difficult to advance because of the insurmountable difficulties in understanding the identity or differentiation of many of the holotypes. DNA barcodes greatly helped us to determine the morphological features which play an important role in species discrimination vs. those that are likely to merely reflect intraspecific variation. The procedure employed here has clarified the taxonomic status of many component taxa so that an escape from this taxonomic quagmire now appears possible. Although this case is extreme, it is certainly not unique. The taxonomy of many groups of animals with limited or poorly understood morphological differentiation may be repaired with the adoption of DNA‐based tools, optimally in combination with other sources of taxonomic information.

M.M. and L.K. contributed the original idea; M.K., M.M. and L.K. designed the research; M.K., M.M., L.K., S.P. and P.H. performed the research; P.H. contributed reagents and analytical tools; M.K., M.M. and L.K. analysed the data; M.M., M.K., L.K., S.P. and P.H. wrote the manuscript.

## Data accessibility

DNA sequences: GenBank Accession nos are provided in Table S3 (Supporting Information).

The full specimen and sequence data are available through a public dataset DS‐ELADIS at BOLD (www.boldsystems.org), DOI: http://dx.doi.org/10.5883/DS-ELADIS.

The following data are available online in Supporting Information files S1–S16: Checklist of species of *Elachista dispunctella* complex, type specimen information, specimen data, ABGD source code, XML files for executing BEAST, R code, schematic depiction of male and female genitalia of *E. dispunctella* complex, FASTA MSA for OTU delineation and type association, a graph showing the number of OTUs plotted against *P*‐values, neighbour‐joining, maximum likelihood and Bayesian trees and blog logic classification formulas.

## Supporting information


**Appendix S1** Checklist of species of *Elachista dispunctella* complex

**Appendix S4** ABGD source code

**Appendix S5** XML file for executing BEAST (OTU delineation)

**Appendix S6** R code

**Appendix S9** XML file for executing BEAST (type association)

**Appendix S10** FASTA MSA for OTU delineation

**Appendix S15** FASTA MSA for type association

**Appendix S16** BLOG logic classification formulas

**Figure S7** Schematic depiction of male genitalia of *E. dispunctella* complex

**Figure S8** Schematic depiction of female genitalia of *E. dispunctella* complex

**Figure S11** The number of OTUs plotted against *P*‐values

**Figure S12** NJ tree

**Figure S13** ML tree

**Figure S14** Bayesian tree

**Table S2** Type specimen information of species of *E. dispunctella* complex

**Table S3** Specimen data
