# Supplementary material for: One species in eight: DNA barcodes from type specimens resolve a taxonomic quagmire
Source: Mol Ecol Resour. 2015 Jan 5;15(4):967–84. doi: 10.1111/1755-0998.12361 (PMC4964951; doi:10.1111/1755-0998.12361)
Supplement: Supplementary file 1 — Appendix S1 Checklist of species of Elachista dispunctella complex [file MEN-15-967-s001.docx]

Supplement/Appendix X

List of nominal species attributed to the *Elachista dispunctella* group with basic data on their description and type localities

***Elachista amparoae*** Traugott-Olsen, 1992. Type locality: Spain: Albacete, Paterna del Madera.

***Elachista andorraensis*** Traugott-Olsen, 1988. Type locality: Andorra: Port de Cábus.

***Elachista anitella*** Traugott-Olsen, 1985. Type locality: Spain: Andalucia, Sierra Nevada, Cam. d. Veleta.

***Elachista arenbergeri*** Traugott-Olsen, 1988. Type locality: Tunisia: 20 km S. of Hammamet, Sebjha Si, Kralifa.

***Elachista baldizzonella*** Traugott-Olsen, 1985. Type locality: Spain: Andalucia, Sierra Nevada, Cam. d. Veleta.

***Elachista bazaella*** Traugott-Olsen, 1992. Type locality: Spain: Andalucia, Sierra Nevada, Cam. d. Veleta.

***Elachista bengtssoni*** Traugott-Olsen, 1992. Type locality: Spain: Alicante, Torrevieja, Lago Jardín.

***Elachista berndtiella*** Traugott-Olsen, 1985. Type locality: Spain: Andalucía, Sierra Nevada, Cam. d. Veleta.

***Elachista blancella*** Traugott-Olsen, 1992. Type locality: Turkey: Prov. Ankara, 20 km NW Kizilcahamen.

***Elachista bustilloi*** Traugott-Olsen, 1988. Type locality: Spain: Madrid, Campo Real.

***Elachista cahorsensis*** Traugott-Olsen, 1992. Type locality: Germany: Kyffhäuser, Kattenberg.

***Elachista casascoensis*** Traugott-Olsen, 1992. Type locality: Italy: Piemonte, Val Curone, Poggio di Casasco.

***Elachista catalunella*** Traugott-Olsen, 1992. Type locality: Spain: Andalucía, Prov. Málaga, Camino de Ojén.

***Elachista clintoni*** Traugott-Olsen, 1992. Type locality: France: Cannes.

***Elachista contisella*** Traugott-Olsen, 1922. Type locality: France: Contis.

***Elachista cuencaensis*** Traugott-Olsen, 1992. Type locality: Spain: Cuenca, Paracuellos.

***Elachista dalmatiensis*** Traugott-Olsen, 1992. Type locality: Dalmatia [Croatia], Dubrovnik.

***Elachista deresyensis*** Traugott-Olsen, 1988. Type locality: Turkey [Syria]: Shar Deresy.

***Elachista disemiella*** Zeller, 1847. Type locality: Italy: Sicilia, Messina.

***Elachista dispunctella*** (Duponcel, 1842 [1843]. Type locality: [East Austria].

***Elachista elsaella*** Traugott-Olsen, 1988: 302. Type locality: Sweden: Småland, Högsby.

***Elachista galactitella*** (Eversmann, 1844). Type locality: Russia: Orenburg.

***Elachista gerdmaritella*** Traugott-Olsen, 1992. Type locality: Spain: Andalucía, Prov. Málaga, Camino de Ojén.

***Elachista gielisi*** Traugott-Olsen, 1992. Type locality: Spain: Murcia, La Marta.

***Elachista glaseri*** Traugott-Olsen, 1992. Type locality: Spain: Málaga, Alhama de Murcia.

***Elachista grandella*** Traugott-Olsen, 1992. Type locality: Austria: Pfaffenberg, Porta Hungarica.

***Elachista gregori*** Traugott-Olsen, 1988. Type locality: Austria: Hainburg Berge.

***Elachista hallini*** Traugott-Olsen, 1992. Type locality: Austria: NE Dürnstein, Wachau.

***Elachista hispanica*** Traugott-Olsen, 1992. Type locality: Spain: Catalonia, Collado de Falset.

***Elachista imbi*** Traugott-Olsen, 1992. Type locality: Austria: Hundesheimer Berg, Porta Hungarica.

***Elachista intrigella*** Traugott-Olsen, 1992. Type locality: Austria: Glaslauterriegel, Gumpoldskirchen.

***Elachista karsholti*** Traugott-Olsen, 1992. Type locality: Austria: Glaslauterriegel, Gumpoldskirchen.

***Elachista lerauti*** Traugott-Olsen, 1992. Type locality: France: Milly-La-Forêt.

***Elachista louiseae*** Traugott-Olsen, 1992. Type locality: Spain: Andalucía, Sierra Nevada, Strada Veleta.

***Elachista luqueti*** Traugott-Olsen, 1992. Type locality: France: Draguignan.

***Elachista maboulella*** Chrétien, 1915. Type locality: Algeria: Biskra, Gafsa.

***Elachista madridensis*** Traugott-Olsen, 1992. Type locality: Spain: Madrid, Campo Real.

***Elachista mannella*** Traugott-Olsen, 1992. Type locality: Austria.

***Elachista michelseni*** Traugott-Olsen, 1992. Type locality: Tunisia: Nefta.

***Elachista minusculella*** Traugott-Olsen, 1992. Type locality: Turkey: 50 km E Istanbul.

***Elachista moroccoensis*** Traugott-Olsen, 1992: 235. Type locality: Morocco: Tinerhin.

***Elachista multipunctella*** Traugott-Olsen, 1992. Type locality: Austria: Porta Hungarica, Hundsheimer Berg.

***Elachista nielspederi*** Traugott-Olsen, 1992. Type locality: Austria: Hundsheimer Berg, Porta Hungarica.

***Elachista occidentella*** Traugott-Olsen, 1992. Type locality: Portugal: Capo de Roca.

***Elachista olemartini*** Traugott-Olsen, 1992. Type locality: Tunisia: Nefta.

***Elachista oukaimedenensis*** Traugott-Olsen, 1988. Type locality: Morocco: High Atlas, 4 km E. Oukaimeden.

***Elachista parvula*** Parenti, 1978. Type locality: Italy: Liguria, Noli.

***Elachista pocopunctella*** Traugott-Olsen, 1992. Type locality: Austria: Wien, Mödling.

***Elachista povolnyi*** Traugott-Olsen, 1992. Type locality: Austria: Glaslauterriegel, southern Gumpoldskirchen.

***Elachista punctella*** Traugott-Olsen, 1992. Type locality: Austria: Porta Hungarica, Hundsheimer.

***Elachista rikkeae*** Traugott-Olsen, 1992. Type locality: Spain: Granada, Camino Baza-Benamaurel, 15 km from Baza.

***Elachista rissaniensis*** Traugott-Olsen, 1992. Type locality: Morocco: Rissani, Erfoud.

***Elachista senecai*** Traugott-Olsen, 1992. Type locality: Libya: Gharian, Wadi El Hira.

***Elachista skulei*** Traugott-Olsen, 1992. Type locality: Greece: Lakonia, Mt. Taygetos.

***Elachista svenssoni*** Traugott-Olsen, 1988. Type locality: Austria.

***Elachista totanaensis*** Traugott-Olsen, 1992. Type locality: Spain: Alicante, Totana, SE Murcia.

***Elachista toveella*** Traugott-Olsen, 1985. Type locality: Spain: Andalucia, Sierra Nevada, Cam. d. Veleta.

***Elachista tribertiella*** Traugott-Olsen, 1985. Type locality: Spain: Andalucia, Sierra Nevada, Cam. d. Veleta.

***Elachista triseriatella*** Stainton, 1854. Type locality: England: St. Vincent's Rocks, Durdham Downs nr. Bristol.

***Elachista vanderwolfi*** Traugott-Olsen, 1992. Type locality: France: Drôme, La Penne-s-l'Ouvéze.

***Elachista varensis*** Traugott-Olsen, 1992. Type locality: France: Var, 20 km S. St. Tropez, Cap. Cartaya, Plage de l'Escalet.

***Elachista veletaella*** Traugott-Olsen, 1992. Type locality: Spain, Andalucia, Sierra Nevada, Cam. d. Veleta.

***Elachista vivesi*** Traugott-Olsen, 1992. Type locality: Spain: Madrid, Campo Real.

***Elachista wadielhiraensis*** Traugott-Olsen, 1992. Type locality: Libya: Gharian, Wadi El Hira.

|  |
| --- |
|  |
|  |
|  |
|  |
|  |
|  |
|  |
|  |
|  |
|  |
|  |
|  |
|  |
|  |
|  |
|  |
|  |
|  |
|  |
|  |
|  |
|  |
|  |
|  |
|  |
|  |
|  |
|  |
|  |
|  |
|  |
|  |
|  |
|  |
|  |
|  |
|  |
|  |
|  |
|  |
|  |
|  |
|  |
|  |
|  |
|  |
|  |
|  |
|  |
|  |
|  |
|  |
|  |
|  |
|  |
|  |
|  |
