# Supplementary figures and images for: One species in eight: DNA barcodes from type specimens resolve a taxonomic quagmire
Source: Mol Ecol Resour. 2015 Jan 5;15(4):967–84. doi: 10.1111/1755-0998.12361 (PMC4964951; doi:10.1111/1755-0998.12361)

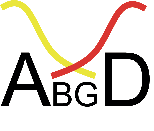

Supplement: Supplementary file 2 — Appendix S4 ABGD source code [file MEN-15-967-s002.tgz › Abgd/abgd.logo.png]

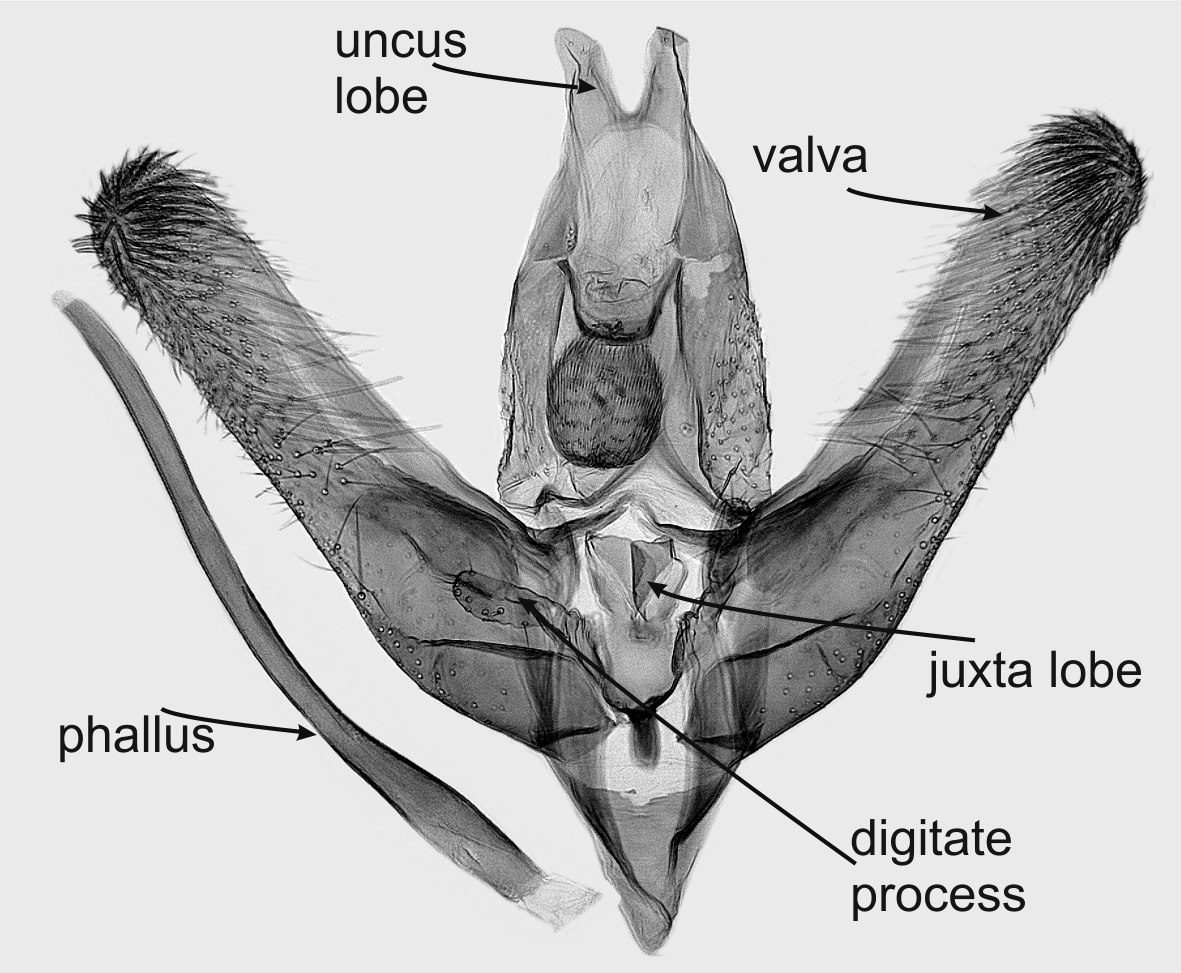

Supplement: Supplementary file 9 — Figure S7 Schematic depiction of male genitalia of E. dispunctella complex [file MEN-15-967-s009.jpg]

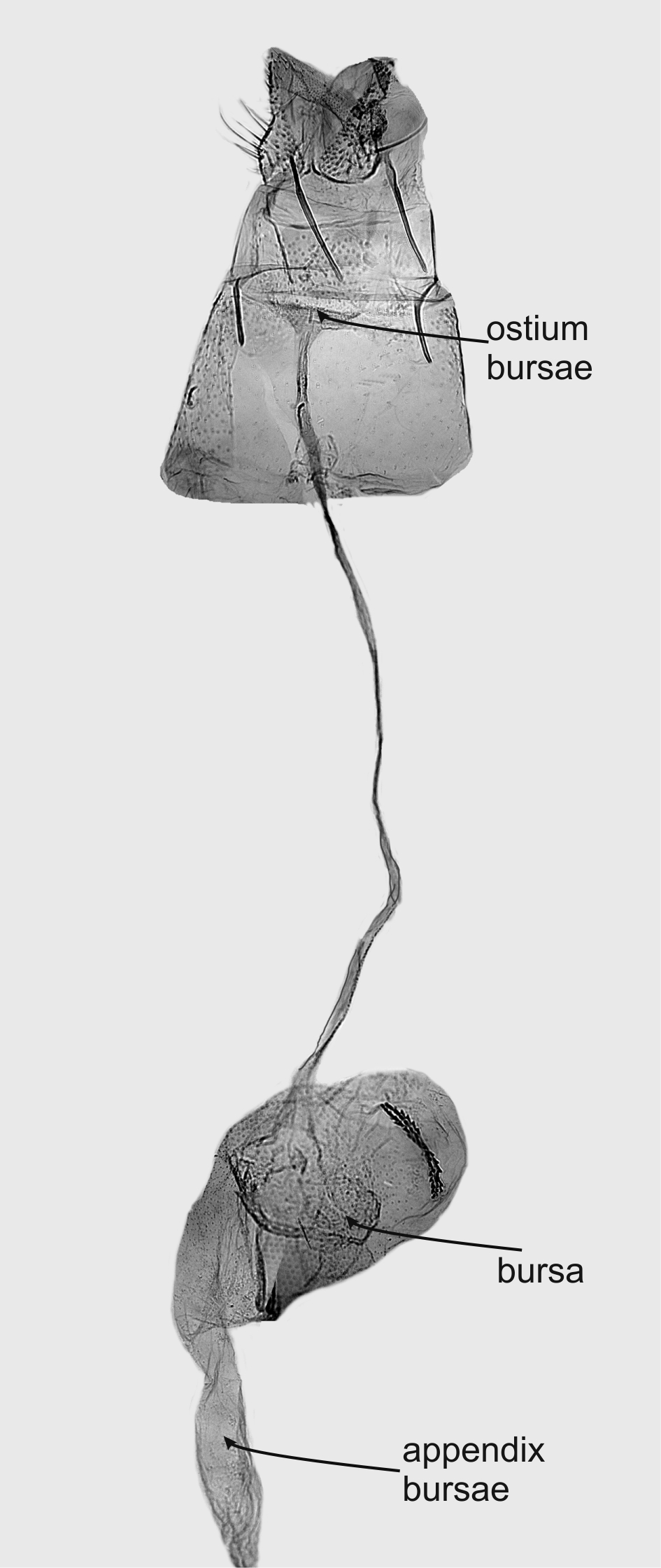

Supplement: Supplementary file 10 — Figure S8 Schematic depiction of female genitalia of E. dispunctella complex [file MEN-15-967-s010.jpg]

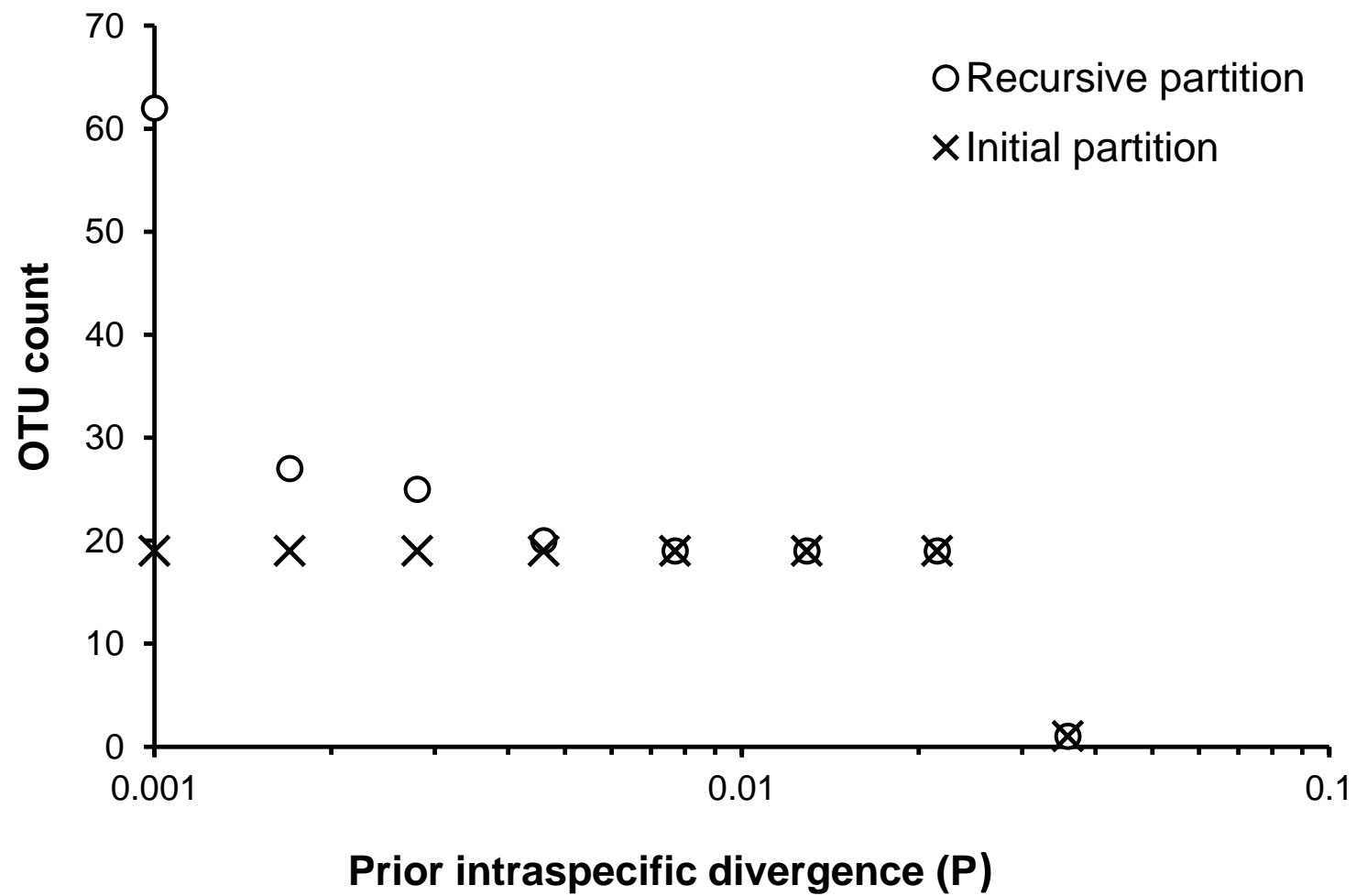

Supplement: Supplementary file 11 — Figure S11 The number of OTUs plotted against P‐values [file MEN-15-967-s011.pdf]

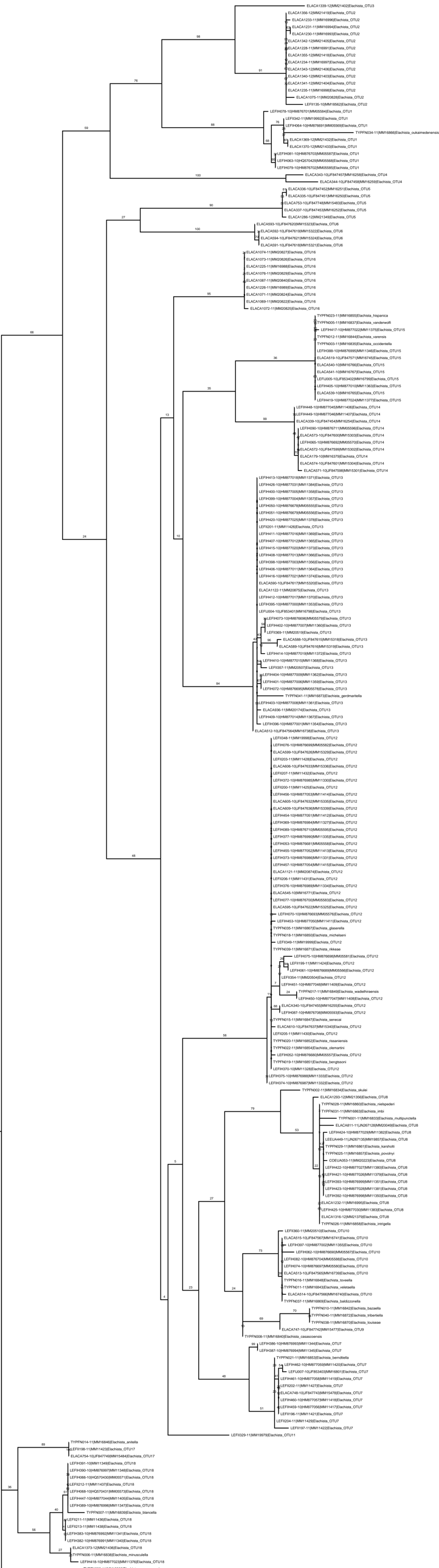

Supplement: Supplementary file 13 — Figure S13 ML tree [file MEN-15-967-s013.pdf]

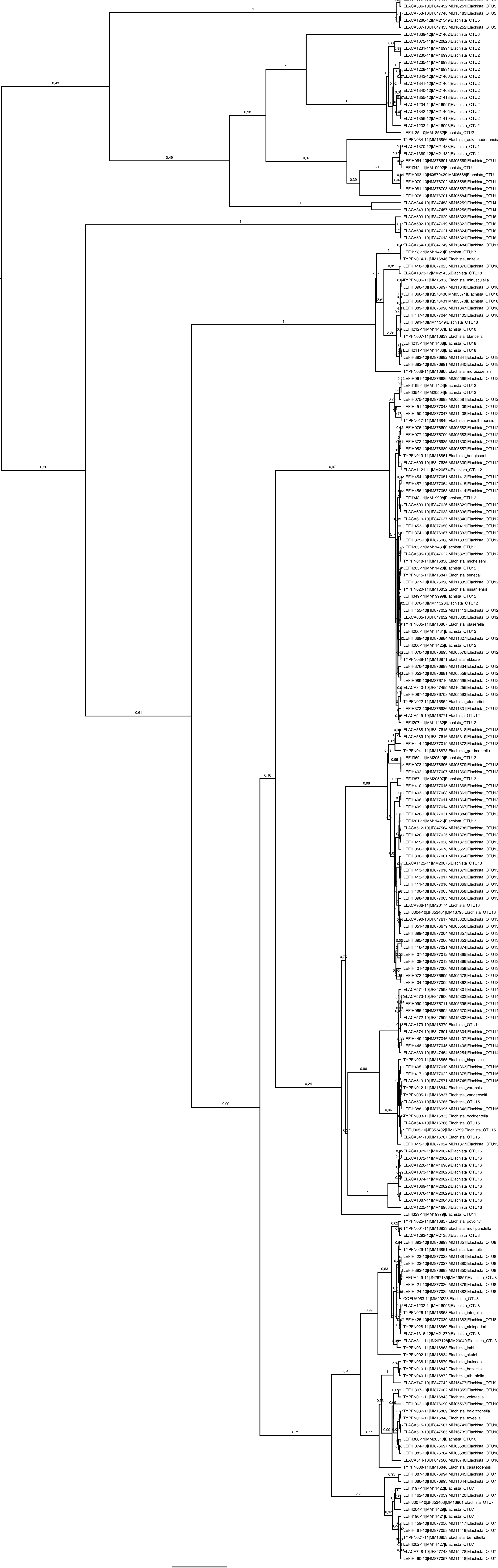

0.0020

Supplement: Supplementary file 14 — Figure S14 Bayesian tree [file MEN-15-967-s014.pdf]
