# Supplementary material for: One species in eight: DNA barcodes from type specimens resolve a taxonomic quagmire
Source: Mol Ecol Resour. 2015 Jan 5;15(4):967–84. doi: 10.1111/1755-0998.12361 (PMC4964951; doi:10.1111/1755-0998.12361)
Supplement: Supplementary file 8 — Appendix S16 BLOG logic classification formulas [file MEN-15-967-s008.docx]

**BLOG logic classification formulas (TRAIN.formulas)**

**654 bp**

CLASS 1: , elachista_otu18

pos450=T , AND , pos465=G

Coverage: , 1.000

False Negative: , 0.000

False Positive: , 0.000

Score (Laplace): , 0.438

FP/TP: , 0.000

CLASS 2: , elachista_otu11

pos367=A , AND , pos450=C

Coverage: , 1.000

False Negative: , 0.000

False Positive: , 0.000

Score (Laplace): , 0.100

FP/TP: , 0.000

CLASS 3: , elachista_otu17

pos546=C , AND , pos645=C

Coverage: , 1.000

False Negative: , 0.000

False Positive: , 0.000

Score (Laplace): , 0.143

FP/TP: , 0.000

CLASS 4: , elachista_otu6

pos156=C , AND , pos399=T

Coverage: , 1.000

False Negative: , 0.000

False Positive: , 0.000

Score (Laplace): , 0.217

FP/TP: , 0.000

CLASS 5: , elachista_otu10

pos138=C , AND , pos432=T

Coverage: , 1.000

False Negative: , 0.000

False Positive: , 0.000

Score (Laplace): , 0.333

FP/TP: , 0.000

CLASS 6: , elachista_otu7

pos42=A , AND , pos450=C

Coverage: , 1.000

False Negative: , 0.000

False Positive: , 0.000

Score (Laplace): , 0.419

FP/TP: , 0.000

CLASS 7: , elachista_otu8

pos42=G

Coverage: , 1.000

False Negative: , 0.000

False Positive: , 0.000

Score (Laplace): , 0.438

FP/TP: , 0.000

CLASS 8: , elachista_otu13

pos411=G

Coverage: , 1.000

False Negative: , 0.000

False Positive: , 0.000

Score (Laplace): , 0.673

FP/TP: , 0.000

CLASS 9: , elachista_otu14

pos411=C , AND , pos450=A

Coverage: , 1.000

False Negative: , 0.000

False Positive: , 0.000

Score (Laplace): , 0.379

FP/TP: , 0.000

CLASS 10: , elachista_otu2

pos363=C , AND , pos411=A

Coverage: , 1.000

False Negative: , 0.000

False Positive: , 0.000

Score (Laplace): , 0.455

FP/TP: , 0.000

CLASS 11: , elachista_otu3

pos411=A , AND , pos546=A

Coverage: , 1.000

False Negative: , 0.000

False Positive: , 0.000

Score (Laplace): , 0.100

FP/TP: , 0.000

CLASS 12: , elachista_otu4

pos273=A

Coverage: , 1.000

False Negative: , 0.000

False Positive: , 0.000

Score (Laplace): , 0.143

FP/TP: , 0.000

CLASS 13: , elachista_otu12

pos385=T , AND , pos399=A

Coverage: , 1.000

False Negative: , 0.000

False Positive: , 0.000

Score (Laplace): , 0.705

FP/TP: , 0.000

CLASS 14: , elachista_otu5

pos78=C

Coverage: , 1.000

False Negative: , 0.000

False Positive: , 0.000

Score (Laplace): , 0.250

FP/TP: , 0.000

CLASS 15: , elachista_otu9

pos301=G

Coverage: , 1.000

False Negative: , 0.000

False Positive: , 0.000

Score (Laplace): , 0.100

FP/TP: , 0.000

CLASS 16: , elachista_otu16

pos276=C , AND , pos618=A

Coverage: , 1.000

False Negative: , 0.000

False Positive: , 0.000

Score (Laplace): , 0.357

FP/TP: , 0.000

CLASS 17: , elachista_otu1

pos546=A , AND , pos636=A

Coverage: , 1.000

False Negative: , 0.000

False Positive: , 0.000

Score (Laplace): , 0.333

FP/TP: , 0.000

CLASS 18: , elachista_otu15

pos432=G

Coverage: , 1.000

False Negative: , 0.000

False Positive: , 0.000

Score (Laplace): , 0.357

FP/TP: , 0.000

CLASS 19: , elachista_otu19

pos513=C , AND , pos633=C

Coverage: , 1.000

False Negative: , 0.000

False Positive: , 0.000

Score (Laplace): , 0.100

FP/TP: , 0.000

**162 bp**

CLASS 1: , elachista_otu18

pos6=T , AND , pos111=C , AND , pos120=C

Coverage: , 1.000

False Negative: , 0.000

False Positive: , 0.000

Score (Laplace): , 0.438

FP/TP: , 0.000

CLASS 2: , elachista_otu11

pos117=C , AND , pos124=C

Coverage: , 1.000

False Negative: , 0.000

False Positive: , 0.000

Score (Laplace): , 0.100

FP/TP: , 0.000

CLASS 3: , elachista_otu17

pos138=T , AND , pos139=T

Coverage: , 1.000

False Negative: , 0.000

False Positive: , 0.000

Score (Laplace): , 0.143

FP/TP: , 0.000

CLASS 4: , elachista_otu6

pos100=C , AND , pos117=T , AND , pos120=C

Coverage: , 1.000

False Negative: , 0.000

False Positive: , 0.000

Score (Laplace): , 0.217

FP/TP: , 0.000

CLASS 5: , elachista_otu10

pos139=C , AND , pos162=C

Coverage: , 1.000

False Negative: , 0.000

False Positive: , 0.000

Score (Laplace): , 0.333

FP/TP: , 0.000

CLASS 6: , elachista_otu7

pos100=T , AND , pos108=T , AND , pos124=C , AND , pos139=T

Coverage: , 1.000

False Negative: , 0.000

False Positive: , 0.000

Score (Laplace): , 0.419

FP/TP: , 0.000

CLASS 7: , elachista_otu8

pos78=G , AND , pos139=C

Coverage: , 1.000

False Negative: , 0.000

False Positive: , 0.000

Score (Laplace): , 0.438

FP/TP: , 0.000

CLASS 8: , elachista_otu13

pos114=C , AND , pos124=C , AND , pos162=T

Coverage: , 1.000

False Negative: , 0.000

False Positive: , 0.000

Score (Laplace): , 0.673

FP/TP: , 0.000

CLASS 9: , elachista_otu14

pos114=C , AND , pos117=C

Coverage: , 1.000

False Negative: , 0.000

False Positive: , 0.000

Score (Laplace): , 0.379

FP/TP: , 0.000

CLASS 10: , elachista_otu2

pos39=C

Coverage: , 1.000

False Negative: , 0.000

False Positive: , 0.000

Score (Laplace): , 0.455

FP/TP: , 0.000

CLASS 11: , elachista_otu3

pos39=T

Coverage: , 1.000

False Negative: , 0.000

False Positive: , 0.000

Score (Laplace): , 0.100

FP/TP: , 0.000

CLASS 12: , elachista_otu4

pos111=A

Coverage: , 1.000

False Negative: , 0.000

False Positive: , 0.000

Score (Laplace): , 0.143

FP/TP: , 0.000

CLASS 13: , elachista_otu12

pos124=T , AND , pos162=C

Coverage: , 1.000

False Negative: , 0.000

False Positive: , 0.000

Score (Laplace): , 0.705

FP/TP: , 0.000

CLASS 14: , elachista_otu5

pos60=T , AND , pos139=C

Coverage: , 1.000

False Negative: , 0.000

False Positive: , 0.000

Score (Laplace): , 0.250

FP/TP: , 0.000

CLASS 15: , elachista_otu9

pos139=G

Coverage: , 1.000

False Negative: , 0.000

False Positive: , 0.000

Score (Laplace): , 0.100

FP/TP: , 0.000

CLASS 16: , elachista_otu16

pos21=T

Coverage: , 1.000

False Negative: , 0.000

False Positive: , 0.000

Score (Laplace): , 0.357

FP/TP: , 0.000

CLASS 17: , elachista_otu1

pos117=A , AND , pos120=T , AND , pos162=T

Coverage: , 1.000

False Negative: , 0.000

False Positive: , 0.000

Score (Laplace): , 0.333

FP/TP: , 0.000

CLASS 18: , elachista_otu15

pos57=T , AND , pos105=G

Coverage: , 1.000

False Negative: , 0.000

False Positive: , 0.000

Score (Laplace): , 0.357

FP/TP: , 0.000

CLASS 19: , elachista_otu19

pos111=C , AND , pos162=C

Coverage: , 1.000

False Negative: , 0.000

False Positive: , 0.000

Score (Laplace): , 0.100

FP/TP: , 0.000

**93 bp**

CLASS 1: , elachista_otu18

pos42=C , AND , pos48=T , AND , pos70=T

Coverage: , 1.000

False Negative: , 0.000

False Positive: , 0.000

Score (Laplace): , 0.438

FP/TP: , 0.000

CLASS 2: , elachista_otu11

pos48=C , AND , pos55=C

Coverage: , 1.000

False Negative: , 0.000

False Positive: , 0.000

Score (Laplace): , 0.100

FP/TP: , 0.000

CLASS 3: , elachista_otu17

pos42=T , AND , pos69=T

Coverage: , 1.000

False Negative: , 0.000

False Positive: , 0.000

Score (Laplace): , 0.143

FP/TP: , 0.000

CLASS 4: , elachista_otu6

pos31=C , AND , pos48=T , AND , pos51=C

Coverage: , 1.000

False Negative: , 0.000

False Positive: , 0.000

Score (Laplace): , 0.217

FP/TP: , 0.000

CLASS 5: , elachista_otu10

pos70=C , AND , pos93=C

Coverage: , 1.000

False Negative: , 0.000

False Positive: , 0.000

Score (Laplace): , 0.333

FP/TP: , 0.000

CLASS 6: , elachista_otu7

pos39=T , AND , pos51=T , AND , pos55=C , AND , pos70=T

Coverage: , 1.000

False Negative: , 0.000

False Positive: , 0.000

Score (Laplace): , 0.419

FP/TP: , 0.000

CLASS 7: , elachista_otu8

pos9=G , AND , pos70=C

Coverage: , 1.000

False Negative: , 0.000

False Positive: , 0.000

Score (Laplace): , 0.438

FP/TP: , 0.000

CLASS 8: , elachista_otu13

pos31=C , AND , pos51=T , AND , pos55=C , AND , pos93=T

Coverage: , 1.000

False Negative: , 0.000

False Positive: , 0.000

Score (Laplace): , 0.673

FP/TP: , 0.000

CLASS 9: , elachista_otu14

pos18=C , AND , pos48=C

Coverage: , 1.000

False Negative: , 0.000

False Positive: , 0.000

Score (Laplace): , 0.379

FP/TP: , 0.000

CLASS 10: , elachista_otu2

pos46=T , AND , pos70=T

Coverage: , 1.000

False Negative: , 0.000

False Positive: , 0.000

Score (Laplace): , 0.455

FP/TP: , 0.000

CLASS 11: , elachista_otu3

pos42=C , AND , pos48=A

Coverage: , 1.000

False Negative: , 0.000

False Positive: , 0.000

Score (Laplace): , 0.100

FP/TP: , 0.000

CLASS 12: , elachista_otu4

pos42=A

Coverage: , 1.000

False Negative: , 0.000

False Positive: , 0.000

Score (Laplace): , 0.143

FP/TP: , 0.000

CLASS 13: , elachista_otu12

pos55=T , AND , pos93=C

Coverage: , 1.000

False Negative: , 0.000

False Positive: , 0.000

Score (Laplace): , 0.705

FP/TP: , 0.000

CLASS 14: , elachista_otu5

pos39=C , AND , pos48=T , AND , pos70=C

Coverage: , 1.000

False Negative: , 0.000

False Positive: , 0.000

Score (Laplace): , 0.250

FP/TP: , 0.000

CLASS 15: , elachista_otu9

pos70=G

Coverage: , 1.000

False Negative: , 0.000

False Positive: , 0.000

Score (Laplace): , 0.100

FP/TP: , 0.000

CLASS 16: , elachista_otu16

pos31=C , AND , pos93=C

Coverage: , 1.000

False Negative: , 0.000

False Positive: , 0.000

Score (Laplace): , 0.357

FP/TP: , 0.000

CLASS 17: , elachista_otu1

pos48=A , AND , pos51=T , AND , pos93=T

Coverage: , 1.000

False Negative: , 0.000

False Positive: , 0.000

Score (Laplace): , 0.333

FP/TP: , 0.000

CLASS 18: , elachista_otu15

pos36=G , AND , pos55=T

Coverage: , 1.000

False Negative: , 0.000

False Positive: , 0.000

Score (Laplace): , 0.357

FP/TP: , 0.000

**54 bp**

CLASS 1: , elachista_otu18

pos3=C , AND , pos9=T , AND , pos31=T

Coverage: , 1.000

False Negative: , 0.000

False Positive: , 0.000

Score (Laplace): , 0.438

FP/TP: , 0.000

CLASS 2: , elachista_otu11

pos16=C , AND , pos42=C

Coverage: , 1.000

False Negative: , 0.000

False Positive: , 0.000

Score (Laplace): , 0.100

FP/TP: , 0.000

CLASS 3: , elachista_otu17

pos16=C , AND , pos30=T

Coverage: , 1.000

False Negative: , 0.000

False Positive: , 0.000

Score (Laplace): , 0.143

FP/TP: , 0.000

CLASS 4: , elachista_otu6

pos51=T

Coverage: , 1.000

False Negative: , 0.000

False Positive: , 0.000

Score (Laplace): , 0.217

FP/TP: , 0.000

CLASS 5: , elachista_otu10

pos31=C , AND , pos54=C

Coverage: , 1.000

False Negative: , 0.000

False Positive: , 0.000

Score (Laplace): , 0.333

FP/TP: , 0.000

CLASS 6: , elachista_otu7

pos6=T , AND , pos12=T , AND , pos16=C , AND , pos31=T

Coverage: , 1.000

False Negative: , 0.000

False Positive: , 0.000

Score (Laplace): , 0.419

FP/TP: , 0.000

CLASS 7: , elachista_otu8

pos16=C , AND , pos31=C , AND , pos54=T

Coverage: , 1.000

False Negative: , 0.000

False Positive: , 0.000

Score (Laplace): , 0.438

FP/TP: , 0.000

CLASS 8: , elachista_otu13

pos6=C , AND , pos9=T , AND , pos54=T

Coverage: , 1.000

False Negative: , 0.000

False Positive: , 0.000

Score (Laplace): , 0.673

FP/TP: , 0.000

CLASS 9: , elachista_otu14

pos12=T , AND , pos42=C

Coverage: , 1.000

False Negative: , 0.000

False Positive: , 0.000

Score (Laplace): , 0.379

FP/TP: , 0.000

CLASS 10: , elachista_otu2

pos6=T , AND , pos48=T

Coverage: , 1.000

False Negative: , 0.000

False Positive: , 0.000

Score (Laplace): , 0.455

FP/TP: , 0.000

CLASS 11: , elachista_otu3

pos6=C , AND , pos48=T

Coverage: , 1.000

False Negative: , 0.000

False Positive: , 0.000

Score (Laplace): , 0.100

FP/TP: , 0.000

CLASS 12: , elachista_otu4

pos15=A

Coverage: , 1.000

False Negative: , 0.000

False Positive: , 0.000

Score (Laplace): , 0.143

FP/TP: , 0.000

CLASS 13: , elachista_otu12

pos16=T , AND , pos54=C

Coverage: , 1.000

False Negative: , 0.000

False Positive: , 0.000

Score (Laplace): , 0.705

FP/TP: , 0.000

CLASS 14: , elachista_otu5

pos9=T , AND , pos16=T , AND , pos31=C

Coverage: , 1.000

False Negative: , 0.000

False Positive: , 0.000

Score (Laplace): , 0.250

FP/TP: , 0.000

CLASS 15: , elachista_otu9

pos31=G

Coverage: , 1.000

False Negative: , 0.000

False Positive: , 0.000

Score (Laplace): , 0.100

FP/TP: , 0.000

CLASS 16: , elachista_otu16

pos6=C , AND , pos54=C

Coverage: , 1.000

False Negative: , 0.000

False Positive: , 0.000

Score (Laplace): , 0.357

FP/TP: , 0.000

CLASS 17: , elachista_otu1

pos9=A , AND , pos12=T , AND , pos54=T

Coverage: , 1.000

False Negative: , 0.000

False Positive: , 0.000

Score (Laplace): , 0.333

FP/TP: , 0.000

CLASS 18: , elachista_otu15

pos9=T , AND , pos16=T , AND , pos31=T , AND , pos54=T

Coverage: , 1.000

False Negative: , 0.000

False Positive: , 0.000

Score (Laplace): , 0.357

FP/TP: , 0.000
