# Supplementary material for: One species in eight: DNA barcodes from type specimens resolve a taxonomic quagmire
Source: Mol Ecol Resour. 2015 Jan 5;15(4):967–84. doi: 10.1111/1755-0998.12361 (PMC4964951; doi:10.1111/1755-0998.12361)
Supplement: Supplementary file 12 — Figure S12 NJ tree [file MEN-15-967-s012.pdf]

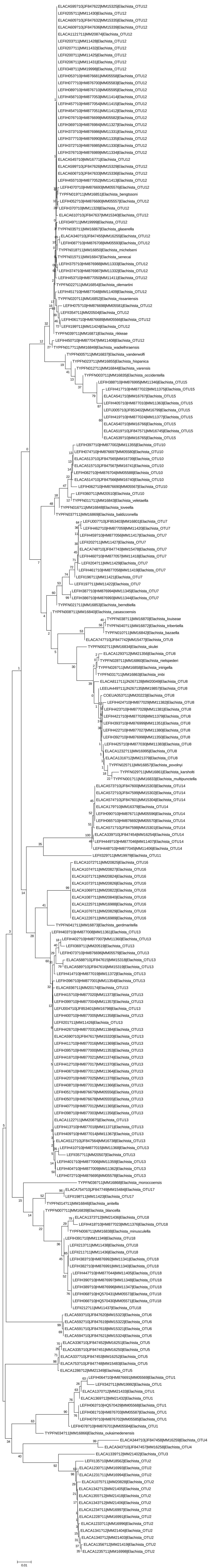
ELACA595?10|JF847622|MM15325|Elachista_OTU12
LEFII205?11|MM11430|Elachista_OTU12
ELACA605?10|JF847632|MM15335|Elachista_OTU12
ELACA609?10|JF847636|MM15339|Elachista_OTU12
ELACA1121?11|MM20874|Elachista_OTU12
LEFII203?11|MM11428|Elachista_OTU12
LEFII207?11|MM11432|Elachista_OTU12
LEFII200?11|MM11425|Elachista_OTU12
LEFII206?11|MM11431|Elachista_OTU12
LEFII348?11|MM19998|Elachista_OTU12
LEFIH053?10|HM876681|MM05558|Elachista_OTU12
LEFIH077?10|HM876700|MM05583|Elachista_OTU12
LEFIH089?10|HM876710|MM05595|Elachista_OTU12
LEFIH456?10|HM877053|MM11414|Elachista_OTU12
LEFIH457?10|HM877054|MM11415|Elachista_OTU12
LEFIH454?10|HM877051|MM11412|Elachista_OTU12
LEFIH076?10|HM876699|MM05582|Elachista_OTU12
LEFIH369?10|HM876984|MM11327|Elachista_OTU12
LEFIH373?10|HM876986|MM11331|Elachista_OTU12
LEFIH377?10|HM876990|MM11335|Elachista_OTU12
LEFIH372?10|HM876985|MM11330|Elachista_OTU12
LEFIH376?10|HM876989|MM11334|Elachista_OTU12
ELACA545?10|MM16771|Elachista_OTU12
ELACA599?10|JF847626|MM15329|Elachista_OTU12
ELACA606?10|JF847633|MM15336|Elachista_OTU12
LEFIH455?10|HM877052|MM11413|Elachista_OTU12
LEFIH070?10|HM876693|MM05576|Elachista_OTU12
TYPFN019?11|MM16851|Elachista_bengtssoni
LEFIH052?10|HM876680|MM05557|Elachista_OTU12
LEFIH370?10|MM11328|Elachista_OTU12
ELACA610?10|JF847637|MM15340|Elachista_OTU12
LEFII349?11|MM19999|Elachista_OTU12
TYPFN035?11|MM16867|Elachista_glaserella
ELACA340?10|JF847455|MM16255|Elachista_OTU12
LEFIH087?10|HM876708|MM05593|Elachista_OTU12
TYPFN018?11|MM16850|Elachista_michelseni
TYPFN015?11|MM16847|Elachista_senecai
LEFIH375?10|HM876988|MM11333|Elachista_OTU12
LEFIH374?10|HM876987|MM11332|Elachista_OTU12
LEFIH453?10|HM877050|MM11411|Elachista_OTU12
TYPFN022?11|MM16854|Elachista_olemartini
LEFIH451?10|HM877048|MM11409|Elachista_OTU12
TYPFN020?11|MM16852|Elachista_rissaniensis
LEFIH075?10|HM876698|MM05581|Elachista_OTU12
LEFII354?11|MM20504|Elachista_OTU12
LEFIH061?10|HM876689|MM05566|Elachista_OTU12
LEFII199?11|MM11424|Elachista_OTU12
TYPFN039?11|MM16871|Elachista_rikkeae
LEFIH450?10|HM877047|MM11408|Elachista_OTU12
TYPFN017?11|MM16849|Elachista_wadielhiraensis
TYPFN005?11|MM16837|Elachista_vanderwolfi
TYPFN023?11|MM16855|Elachista_hispanica
TYPFN012?11|MM16844|Elachista_varensis
TYPFN003?11|MM16835|Elachista_occidentella
LEFIH388?10|HM876995|MM11346|Elachista_OTU15
LEFIH417?10|HM877022|MM11375|Elachista_OTU15
ELACA541?10|MM16767|Elachista_OTU15
LEFIH405?10|HM877010|MM11363|Elachista_OTU15
LEFIJ005?10|JF853402|MM16799|Elachista_OTU15
LEFIH419?10|HM877024|MM11377|Elachista_OTU15
ELACA540?10|MM16766|Elachista_OTU15
ELACA519?10|JF847571|MM16745|Elachista_OTU15
ELACA539?10|MM16765|Elachista_OTU15
LEFIH397?10|HM877002|MM11355|Elachista_OTU10
LEFIH074?10|HM876697|MM05580|Elachista_OTU10
ELACA513?10|JF847565|MM16739|Elachista_OTU10
ELACA515?10|JF847567|MM16741|Elachista_OTU10
LEFIH082?10|HM876704|MM05588|Elachista_OTU10
ELACA514?10|JF847566|MM16740|Elachista_OTU10
LEFIH062?10|HM876690|MM05567|Elachista_OTU10
LEFII360?11|MM20510|Elachista_OTU10
TYPFN011?11|MM16843|Elachista_veletaella
TYPFN016?11|MM16848|Elachista_toveella
TYPFN037?11|MM16869|Elachista_baldizzonella
LEFIJ007?10|JF853403|MM16801|Elachista_OTU7
LEFIH462?10|HM877059|MM11420|Elachista_OTU7
LEFIH459?10|HM877056|MM11417|Elachista_OTU7
LEFII202?11|MM11427|Elachista_OTU7
ELACA748?10|JF847743|MM15478|Elachista_OTU7
LEFIH460?10|HM877057|MM11418|Elachista_OTU7
LEFII204?11|MM11429|Elachista_OTU7
LEFIH461?10|HM877058|MM11419|Elachista_OTU7
LEFII196?11|MM11421|Elachista_OTU7
LEFII197?11|MM11422|Elachista_OTU7
LEFIH387?10|HM876994|MM11345|Elachista_OTU7
LEFIH386?10|HM876993|MM11344|Elachista_OTU7
TYPFN021?11|MM16853|Elachista_berndtiella
TYPFN008?11|MM16840|Elachista_casascoensis
TYPFN038?11|MM16870|Elachista_louiseae
TYPFN040?11|MM16872|Elachista_tribertiella
TYPFN010?11|MM16842|Elachista_bazaella
ELACA747?10|JF847742|MM15477|Elachista_OTU9
TYPFN002?11|MM16834|Elachista_skulei
ELACA1293?12|MM21356|Elachista_OTU8
TYPFN028?11|MM16860|Elachista_nielspederi
TYPFN026?11|MM16858|Elachista_intrigella
TYPFN031?11|MM16863|Elachista_imbi
ELACA811?11|JN267128|MM20049|Elachista_OTU8
LEEUA449?11|JN267135|MM19857|Elachista_OTU8
COEUA053?11|MM20223|Elachista_OTU8
LEFIH424?10|HM877029|MM11382|Elachista_OTU8
LEFIH423?10|HM877028|MM11381|Elachista_OTU8
LEFIH421?10|HM877026|MM11379|Elachista_OTU8
LEFIH393?10|HM876999|MM11351|Elachista_OTU8
LEFIH422?10|HM877027|MM11380|Elachista_OTU8
LEFIH392?10|HM876998|MM11350|Elachista_OTU8
LEFIH425?10|HM877030|MM11383|Elachista_OTU8
ELACA1232?11|MM16995|Elachista_OTU8
ELACA1316?12|MM21379|Elachista_OTU8
TYPFN025?11|MM16857|Elachista_povolnyi
TYPFN029?11|MM16861|Elachista_karsholti
TYPFN001?11|MM16833|Elachista_multipunctella
ELACA573?10|JF847600|MM15303|Elachista_OTU14
ELACA572?10|JF847599|MM15302|Elachista_OTU14
ELACA574?10|JF847601|MM15304|Elachista_OTU14
ELACA179?10|MM16379|Elachista_OTU14
LEFIH090?10|HM876711|MM05596|Elachista_OTU14
LEFIH065?10|HM876692|MM05570|Elachista_OTU14
ELACA571?10|JF847598|MM15301|Elachista_OTU14
ELACA339?10|JF847454|MM16254|Elachista_OTU14
LEFIH449?10|HM877046|MM11407|Elachista_OTU14
LEFIH448?10|HM877045|MM11406|Elachista_OTU14
LEFII329?11|MM19979|Elachista_OTU11
ELACA1072?11|MM20825|Elachista_OTU16
ELACA1074?11|MM20827|Elachista_OTU16
ELACA1071?11|MM20824|Elachista_OTU16
ELACA1073?11|MM20826|Elachista_OTU16
ELACA1069?11|MM20822|Elachista_OTU16
ELACA1087?11|MM20840|Elachista_OTU16
ELACA1225?11|MM16988|Elachista_OTU16
ELACA1076?11|MM20829|Elachista_OTU16
ELACA1226?11|MM16989|Elachista_OTU16
TYPFN041?11|MM16873|Elachista_gerdmaritella
LEFIH403?10|HM877008|MM11361|Elachista_OTU13
LEFIH402?10|HM877007|MM11360|Elachista_OTU13
LEFII369?11|MM20519|Elachista_OTU13
LEFIH073?10|HM876696|MM05579|Elachista_OTU13
ELACA588?10|JF847615|MM15318|Elachista_OTU13
ELACA589?10|JF847616|MM15319|Elachista_OTU13
LEFIH414?10|HM877019|MM11372|Elachista_OTU13
LEFIH396?10|HM877001|MM11354|Elachista_OTU13
ELACA936?11|MM20174|Elachista_OTU13
LEFIH415?10|HM877020|MM11373|Elachista_OTU13
LEFIH399?10|HM877004|MM11357|Elachista_OTU13
LEFIJ004?10|JF853401|MM16798|Elachista_OTU13
LEFIH400?10|HM877005|MM11358|Elachista_OTU13
LEFII201?11|MM11426|Elachista_OTU13
LEFIH426?10|HM877031|MM11384|Elachista_OTU13
ELACA590?10|JF847617|MM15320|Elachista_OTU13
LEFIH411?10|HM877016|MM11369|Elachista_OTU13
LEFIH395?10|HM877000|MM11353|Elachista_OTU13
LEFIH416?10|HM877021|MM11374|Elachista_OTU13
LEFIH412?10|HM877017|MM11370|Elachista_OTU13
LEFIH406?10|HM877011|MM11364|Elachista_OTU13
LEFIH420?10|HM877025|MM11378|Elachista_OTU13
LEFIH408?10|HM877013|MM11366|Elachista_OTU13
LEFIH051?10|HM876679|MM05556|Elachista_OTU13
LEFIH050?10|HM876678|MM05555|Elachista_OTU13
LEFIH407?10|HM877012|MM11365|Elachista_OTU13
LEFIH398?10|HM877003|MM11356|Elachista_OTU13
ELACA1122?11|MM20875|Elachista_OTU13
LEFIH413?10|HM877018|MM11371|Elachista_OTU13
LEFIH409?10|HM877014|MM11367|Elachista_OTU13
ELACA512?10|JF847564|MM16738|Elachista_OTU13
LEFIH410?10|HM877015|MM11368|Elachista_OTU13
LEFII357?11|MM20507|Elachista_OTU13
LEFIH401?10|HM877006|MM11359|Elachista_OTU13
LEFIH404?10|HM877009|MM11362|Elachista_OTU13
LEFIH072?10|HM876695|MM05578|Elachista_OTU13
TYPFN036?11|MM16868|Elachista_moroccoensis
ELACA754?10|JF847749|MM15484|Elachista_OTU17
LEFII198?11|MM11423|Elachista_OTU17
TYPFN014?11|MM16846|Elachista_anitella
TYPFN007?11|MM16839|Elachista_blancella
ELACA1373?12|MM21436|Elachista_OTU18
LEFIH418?10|HM877023|MM11376|Elachista_OTU18
TYPFN006?11|MM16838|Elachista_minusculella
LEFIH391?10|MM11349|Elachista_OTU18
LEFII213?11|MM11438|Elachista_OTU18
LEFII211?11|MM11436|Elachista_OTU18
LEFIH383?10|HM876992|MM11341|Elachista_OTU18
LEFIH382?10|HM876991|MM11340|Elachista_OTU18
LEFIH447?10|HM877044|MM11405|Elachista_OTU18
LEFIH390?10|HM876997|MM11348|Elachista_OTU18
LEFIH389?10|HM876996|MM11347|Elachista_OTU18
LEFIH068?10|HQ570431|MM05573|Elachista_OTU18
LEFIH066?10|HQ570430|MM05571|Elachista_OTU18
LEFII212?11|MM11437|Elachista_OTU18
ELACA593?10|JF847620|MM15323|Elachista_OTU6
ELACA592?10|JF847619|MM15322|Elachista_OTU6
ELACA591?10|JF847618|MM15321|Elachista_OTU6
ELACA594?10|JF847621|MM15324|Elachista_OTU6
ELACA336?10|JF847452|MM16251|Elachista_OTU5
ELACA335?10|JF847451|MM16250|Elachista_OTU5
ELACA337?10|JF847453|MM16252|Elachista_OTU5
ELACA753?10|JF847748|MM15483|Elachista_OTU5
ELACA1286?12|MM21349|Elachista_OTU5
LEFIH064?10|HM876691|MM05569|Elachista_OTU1
LEFII342?11|MM19992|Elachista_OTU1
ELACA1370?12|MM21433|Elachista_OTU1
ELACA1369?12|MM21432|Elachista_OTU1
LEFIH063?10|HQ570429|MM05568|Elachista_OTU1
LEFIH081?10|HM876703|MM05587|Elachista_OTU1
LEFIH079?10|HM876702|MM05585|Elachista_OTU1
LEFIH078?10|HM876701|MM05584|Elachista_OTU1
TYPFN034?11|MM16866|Elachista_oukaimedenensis
ELACA344?10|JF847458|MM16259|Elachista_OTU4
ELACA343?10|JF847457|MM16258|Elachista_OTU4
ELACA1339?12|MM21402|Elachista_OTU3
LEFII135?10|MM18562|Elachista_OTU2
ELACA1230?11|MM16993|Elachista_OTU2
ELACA1231?11|MM16994|Elachista_OTU2
ELACA1075?11|MM20828|Elachista_OTU2
ELACA1342?12|MM21405|Elachista_OTU2
ELACA1355?12|MM21418|Elachista_OTU2
ELACA1343?12|MM21406|Elachista_OTU2
ELACA1234?11|MM16997|Elachista_OTU2
ELACA1228?11|MM16991|Elachista_OTU2
ELACA1233?11|MM16996|Elachista_OTU2
ELACA1341?12|MM21404|Elachista_OTU2
ELACA1340?12|MM21403|Elachista_OTU2
ELACA1356?12|MM21419|Elachista_OTU2
ELACA1235?11|MM16998|Elachista_OTU2
0.01
