# Supplementary material for: One species in eight: DNA barcodes from type specimens resolve a taxonomic quagmire
Source: Mol Ecol Resour. 2015 Jan 5;15(4):967–84. doi: 10.1111/1755-0998.12361 (PMC4964951; doi:10.1111/1755-0998.12361)
Supplement: Supplementary file 16 — Table S3 Specimen data [file MEN-15-967-s016.pdf]

Sample ID	Species	BIN	Seq. Length	GenBank Accession	Sex	Type status	Collectors	Collection Date	Country	Locality
MM05555	Elachista OTU13	BOLD:AAB3258	658	HM876678	M		Jukka Tabell	22-May-2009	Spain	Comunidad Valenciana, Alicante, Albatera 8, 5 km NNW
MM05556	Elachista OTU13	BOLD:AAB3258	658	HM876679	M		Jukka Tabell	22-May-2009	Spain	Comunidad Valenciana, Alicante, Albatera 8, 5 km NNW
MM05557	Elachista OTU12	BOLD:AAB2139	658	HM876680	M		Jukka Tabell	21-Apr-2009	Spain	Comunidad Valenciana, Alicante, Torrevieja
MM05558	Elachista OTU12	BOLD:AAB2139	658	HM876681	M		Jukka Tabell	21-Apr-2009	Spain	Comunidad Valenciana, Alicante, Torrevieja
MM05566	Elachista OTU12	BOLD:AAB2139	658	HM876689	M		Jukka Tabell	24-May-2009	Spain	Comunidad Valenciana, Alicante, Albatera 7 km NNW
MM05567	Elachista OTU10	BOLD:AAI6215	658	HM876690	M		Jukka Tabell	13-Apr-2009	Spain	Comunidad Valenciana, Alicante, San Miguel 3 km SW
MM05568	Elachista OTU1	BOLD:AAI6216	658	HQ570429	F		Jukka Tabell	01-May-2009	Spain	Comunidad Valenciana, Alicante, San Miguel 3 km E
MM05569	Elachista OTU1	BOLD:AAI6216	658	HM876691	M		Jukka Tabell	12-May-2009	Spain	Comunidad Valenciana, Alicante, Albatera 11km NNW
MM05570	Elachista OTU14	BOLD:AAB2137	658	HM876692	F		Jukka Tabell	30-May-2009	Spain	Catalonia, Barcelona, Sitges 10 km NW
MM05571	Elachista OTU18	BOLD:AAB2133	658	HQ570430	M		Jari Junnilainen	26-Apr-2006	Greece	West Macedonia, Kozani
MM05573	Elachista OTU18	BOLD:AAB2133	658	HQ570431	M		Jari Junnilainen	26-Apr-2006	Greece	West Macedonia, Kozani
MM05576	Elachista OTU12	BOLD:AAB2139	538	HM876693	M		Jukka Tabell	24-May-2009	Spain	Comunidad Valenciana, Alicante, Albatera 7 km NNW
MM05578	Elachista OTU13	BOLD:AAB3258	658	HM876695	M		Jukka Tabell	12-May-2009	Spain	Comunidad Valenciana, Alicante, Albatera 11km NNW
MM05579	Elachista OTU13	BOLD:AAB3258	658	HM876696	M		Jukka Tabell	02-May-2009	Spain	Murcia, Sucina 7 km NW
MM05580	Elachista OTU10	BOLD:AAI6215	658	HM876697	M		Jukka Tabell	23-Apr-2009	Spain	Comunidad Valenciana, Alicante, San Miguel 3 km SW
MM05581	Elachista OTU12	BOLD:AAB2139	658	HM876698	M		Jukka Tabell	29-Apr-2009	Spain	Comunidad Valenciana, Alicante, Torremendo 3, 5 km NW
MM05582	Elachista OTU12	BOLD:AAB2139	658	HM876699	M		Jukka Tabell	05-May-2009	Spain	Comunidad Valenciana, Alicante, Torremendo 3, 8 km NW
MM05583	Elachista OTU12	BOLD:AAB2139	658	HM876700	M		Jukka Tabell	18-Mar-2010	Spain	Murcia, Los Belones 1 km SE
MM05584	Elachista OTU1	BOLD:AAI6216	658	HM876701	M		Jukka Tabell	07-May-2009	Spain	Comunidad Valenciana, Alicante, San Miguel 3 km SW
MM05585	Elachista OTU1	BOLD:AAI6216	658	HM876702	M		Jukka Tabell	01-May-2009	Spain	Comunidad Valenciana, Alicante, San Miguel 3 km SW
MM05587	Elachista OTU1	BOLD:AAI6216	658	HM876703	M		Jukka Tabell	18-Apr-2009	Spain	Comunidad Valenciana, Alicante, San Miguel 3 km E
MM05588	Elachista OTU10	BOLD:AAI6215	635	HM876704	M		Jukka Tabell	16-Apr-2009	Spain	Comunidad Valenciana, Alicante, San Miguel 3 km SW
MM05593	Elachista OTU12	BOLD:AAB2139	658	HM876708	M		Jukka Tabell	em. 06-Apr-2010	Spain	Comunidad Valenciana, Alicante, San Miguel 3 km SW
MM05595	Elachista OTU12	BOLD:AAB2139	658	HM876710	F		Jukka Tabell	em. 06-Apr-2010	Spain	Murcia, Los Belones 1 km SE
MM05596	Elachista OTU14	BOLD:AAB2137	658	HM876711	F		Jukka Tabell	em. 05-Apr-2010	Spain	Catalonia, Barcelona, Sitges 6 km NW
MM11326	Elachista OTU12		307	KJ130910			H. W. V. D. Wolf	1997	Spain	Murcia, Aquilas
MM11327	Elachista OTU12	BOLD:AAB2139	658	HM876984			H. W. V. D. Wolf	1997	Spain	Murcia, Aquilas
MM11328	Elachista OTU12	BOLD:AAB2139	416	KJ130924			H. W. V. D. Wolf	1995	Spain	Murcia, Aquilas
MM11329	Elachista OTU12		307	KJ130913			H. W. V. D. Wolf	1995	Spain	Murcia, Aquilas
MM11330	Elachista OTU12	BOLD:AAB2139	658	HM876985			H. W. V. D. Wolf	1997	Spain	Andalusia, Almeria, Camping Cabo de Gata
MM11331	Elachista OTU12	BOLD:AAB2139	658	HM876986			Jari Junnilainen	2001	Spain	Andalusia, Almeria
MM11332	Elachista OTU12	BOLD:AAB2139	658	HM876987			Jari Junnilainen	2001	Spain	Andalusia, Almeria
MM11333	Elachista OTU12	BOLD:AAB2139	658	HM876988			Jari Junnilainen	2001	Spain	Andalusia, Almeria
MM11334	Elachista OTU12	BOLD:AAB2139	658	HM876989			C. Gielis, F. K. Gielis	2001	Spain	Andalusia, Almeria, Tabernas
MM11335	Elachista OTU12	BOLD:AAB2139	658	HM876990			C. Gielis, F. K. Gielis	2001	Spain	Andalusia, Almeria, Tabernas
MM11340	Elachista OTU18	BOLD:AAB2133	658	HM876991			Yu Budashkin	1996	Ukraine	Crimea, Karadagh
MM11341	Elachista OTU18	BOLD:AAB2133	658	HM876992			Yu Budashkin	1996	Ukraine	Crimea, Karadagh
MM11343	Elachista OTU7		219	KJ130932			H. W. V. D. Wolf		Germany	Rhld-Pfalz Kaub
MM11344	Elachista OTU7	BOLD:AAB2141	658	HM876993			Kimmo Silvonen	1998	France	Provence-Alpes-Cote d'Azur, Alpes-Maritimes, Vallee de la Vesubie
MM11345	Elachista OTU7	BOLD:AAB2141	658	HM876994			Kimmo Silvonen	1998	France	Provence-Alpes-Cote d'Azur, Alpes-Maritimes, St. Martin de Peille
MM11346	Elachista OTU15	BOLD:AAB3257	571	HM876995			C. Gielis, F. K. Gielis	2001	Spain	Andalusia, Granada, Baza
MM11347	Elachista OTU18	BOLD:AAB2133	658	HM876996			J. -P. Kaitila	2003	Macedonia	Kozani
MM11348	Elachista OTU18	BOLD:AAB2133	658	HM876997			J. -P. Kaitila	2003	Macedonia	Kozani
MM11349	Elachista OTU18	BOLD:AAB2133	571	KJ130899			J. -P. Kaitila	2003	Macedonia	Kozani
MM11350	Elachista OTU8	BOLD:AAB2134	658	HM876998			T. Baran	1998	Poland	Glinki k. Torunia
MM11351	Elachista OTU8	BOLD:AAB2134	658	HM876999			T. Baran	1999	Poland	Glinki n. Torun
MM11353	Elachista OTU13	BOLD:AAB3258	658	HM877000			H. W. V. D. Wolf	1997	Spain	Murcia, Sierra d`Espuna
MM11354	Elachista OTU13	BOLD:AAB3258	658	HM877001			H. W. V. D. Wolf	1997	Spain	Murcia, Sierra d`Espuna
MM11355	Elachista OTU10	BOLD:AAI6215	658	HM877002			H. W. V. D. Wolf	2000	Spain	Murcia, Sierra d`Espuna
MM11356	Elachista OTU13	BOLD:AAB3258	658	HM877003			H. W. V. D. Wolf	1997	Spain	Murcia, Sierra d`Espuna
MM11357	Elachista OTU13	BOLD:AAB3258	658	HM877004			H. W. V. D. Wolf	1997	Spain	Murcia, Aguilas
MM11358	Elachista OTU13	BOLD:AAB3258	658	HM877005			H. W. V. D. Wolf	1997	Spain	Murcia, Aguilas
MM11359	Elachista OTU13	BOLD:AAB3258	658	HM877006			H. W. V. D. Wolf	1997	Spain	Murcia, Sierra d`Espuna
MM11360	Elachista OTU13	BOLD:AAB3258	658	HM877007			Jari Junnilainen	2001	Spain	Andalusia, Granada, Motril
MM11361	Elachista OTU13	BOLD:AAB3258	571	HM877008			Jari Junnilainen	2001	Spain	Andalusia, Granada, Motril
MM11362	Elachista OTU13	BOLD:AAB3258	658	HM877009			Jari Junnilainen	2001	Spain	Andalusia, Granada, Motril
MM11363	Elachista OTU15	BOLD:AAB3257	658	HM877010			Jari Junnilainen	1999	Spain	Andalusia, Granada, Valeta
MM11364	Elachista OTU13	BOLD:AAB3258	658	HM877011			Jari Junnilainen	2001	Spain	Andalusia, Almeria
MM11365	Elachista OTU13	BOLD:AAB3258	658	HM877012			Timo Nupponen, Kari Nupponen	2002	Spain	Andalusia, Malaga, Ronda
MM11366	Elachista OTU13	BOLD:AAB3258	658	HM877013			Jari Junnilainen	2001	Spain	Andalusia, Granada, Motril
MM11367	Elachista OTU13	BOLD:AAB3258	656	HM877014			Jari Junnilainen	2001	Spain	Andalusia, Granada, Motril
MM11368	Elachista OTU13	BOLD:AAB3258	658	HM877015			Jari Junnilainen	2001	Spain	Andalusia, Granada, Motril
MM11369	Elachista OTU13	BOLD:AAB3258	658	HM877016			Jari Junnilainen	2001	Spain	Andalusia, Granada, Motril
MM11370	Elachista OTU13	BOLD:AAB3258	658	HM877017			Jari Junnilainen	2001	Spain	Andalusia, Malaga, Marbella
MM11371	Elachista OTU13	BOLD:AAB3258	656	HM877018			Jari Junnilainen	2001	Spain	Andalusia, Malaga, Marbella
MM11372	Elachista OTU13	BOLD:AAB3258	658	HM877019			Jari Junnilainen	2001	Spain	Andalusia, Almeria
MM11373	Elachista OTU13	BOLD:AAB3258	658	HM877020			Timo Nupponen	2000	Spain	Andalusia, Malaga, Marbella
MM11374	Elachista OTU13	BOLD:AAB3258	658	HM877021			Jari Junnilainen	1999	Spain	Andalusia, Granada, Carataunas
MM11375	Elachista OTU15	BOLD:AAB3257	658	HM877022			H. W. V. D. Wolf	2000	Spain	Aragon, Teruel, Cosa
MM11376	Elachista OTU18	BOLD:ACE9135	658	HM877023			Kimmo Silvonen	1998	France	Provence-Alpes-Cote d'Azur, Alpes-Maritimes, Col de Vence
MM11377	Elachista OTU15	BOLD:AAB3257	658	HM877024			C. Gielis, F. K. Gielis	2001	Spain	Andalusia, Granada, Baza
MM11378	Elachista OTU13	BOLD:AAB3258	658	HM877025			H. W. V. D. Wolf	1995	Spain	Murcia, Aguilas
MM11379	Elachista OTU8	BOLD:AAB2134	658	HM877026			Kari Nupponen	2001	Russia	Volgograd, Ilovla
MM11380	Elachista OTU8	BOLD:AAB2134	658	HM877027			Kari Nupponen	2005	Russia	Volgograd, Frolovo
MM11381	Elachista OTU8	BOLD:AAB2134	658	HM877028			Kari Nupponen	2001	Russia	Volgograd, Ilovla
MM11382	Elachista OTU8	BOLD:AAB2134	658	HM877029			Kari Nupponen	2005	Russia	Orenburg, S-Ural, Orenburg/Burannoe
MM11383	Elachista OTU8	BOLD:AAB2134	658	HM877030			Kari Nupponen	2005	Russia	Orenburg, S-Ural, Orenburg/Burannoe
MM11384	Elachista OTU13	BOLD:AAB3258	658	HM877031			Jari Junnilainen	2004	Spain	Aragon, Los Monegros Caspe
MM11405	Elachista OTU18	BOLD:AAB2133	658	HM877044			Jari Junnilainen	1999	Turkey	Kauseri, Ergiyes Dagi
MM11406	Elachista OTU14	BOLD:AAB2137	621	HM877045			Kari Nupponen	2000	Tunisia	Atlas mnts., Le Kef/ El Ksour
MM11407	Elachista OTU14	BOLD:AAB2137	658	HM877046			Kari Nupponen	2000	Tunisia	Atlas mnts., Makthar
MM11408	Elachista OTU12	BOLD:AAB2139	657	HM877047			Kari Nupponen	2000	Tunisia	Nefta, (Sahara)
MM11409	Elachista OTU12	BOLD:AAB2139	657	HM877048			Kari Nupponen	2000	Tunisia	Nefta, (Sahara)
MM11411	Elachista OTU12	BOLD:AAB2139	658	HM877050			Kari Nupponen	2007	Spain	Aragon, Zaragoza, Los Monegros Gelsa
MM11412	Elachista OTU12	BOLD:AAB2139	658	HM877051			Kari Nupponen	2007	Spain	Aragon, Zaragoza, Los Monegros Gelsa
MM11413	Elachista OTU12	BOLD:AAB2139	658	HM877052			Kari Nupponen	2007	Spain	Aragon, Zaragoza, Los Monegros Gelsa
MM11414	Elachista OTU12	BOLD:AAB2139	658	HM877053			Kari Nupponen	2007	Spain	Aragon, Zaragoza, Los Monegros Gelsa
MM11415	Elachista OTU12	BOLD:AAB2139	658	HM877054			Timo Nupponen	2007	Spain	Aragon, Zaragoza, Monegrillo
MM11417	Elachista OTU7	BOLD:AAB2141	657	HM877056			Kari Nupponen	2008	Spain	Aragon, Teniel, Villalba Baja
MM11418	Elachista OTU7	BOLD:AAB2141	658	HM877057			Kari Nupponen	2008	Spain	Aragon, Teniel, Villalba Baja
MM11419	Elachista OTU7	BOLD:AAB2141	657	HM877058			Kari Nupponen	2008	Spain	Castilla-La Mancha, Cuenca, Fuentes
MM11420	Elachista OTU7	BOLD:AAB2141	658	HM877059			Kari Nupponen	2008	Spain	Castilla-La Mancha, Cuenca, Fuentes
MM11421	Elachista OTU7	BOLD:AAB2141	624	KJ130911			Kari Nupponen	2008	Spain	Castilla-La Mancha, Cuenca, Fuentes
MM11422	Elachista OTU7	BOLD:AAB2141	584	KJ130938			Timo Nupponen	2003	Spain	Andalusia, Granada, Sierra Nevada
MM11423	Elachista OTU17	BOLD:AAP4194	658	KJ130881			Timo Nupponen	2003	Spain	Andalusia, Granada, Sierra Nevada
MM11424	Elachista OTU12	BOLD:AAB2139	658	KJ130887			Timo Nupponen, Kari Nupponen	2002	Spain	Andalusia, Almeria
MM11425	Elachista OTU12	BOLD:AAB2139	658	KJ130921			Kari Nupponen	2004	Spain	Andalusia, Almeria
MM11426	Elachista OTU13	BOLD:AAB3258	658	KJ130914			Timo Nupponen, Kari Nupponen	2002	Spain	Andalusia, Malaga, Ronda
MM11427	Elachista OTU7	BOLD:AAB2141	658	KJ130885			Timo Nupponen	2003	Spain	Andalusia, Granada, Sierra Nevada
MM11428	Elachista OTU12	BOLD:AAB2139	658	KJ130877			Timo Nupponen, Kari Nupponen	2002	Spain	Andalusia, Almeria
MM11429	Elachista OTU7	BOLD:AAB2141	658	KJ130912			Timo Nupponen	2004	Spain	Aragon, Teruel Albarracia
MM11430	Elachista OTU12	BOLD:AAB2139	646	KJ130882			Timo Nupponen	2004	Spain	Aragon, Teruel Albarracia
MM11431	Elachista OTU12	BOLD:AAB2139	658	KJ130942			Kari Nupponen	2004	Spain	Andalusia, Granada, Rio de Baza
MM11432	Elachista OTU12	BOLD:AAB2139	658	KJ130897			Kari Nupponen	2004	Spain	Andalusia, Almeria
MM11436	Elachista OTU18	BOLD:AAB2133	632	KJ130891			Yu Budashkin	1996	Ukraine	Krym, Karadagh
MM11437	Elachista OTU18	BOLD:AAB2133	658	KJ130886			Yu Budashkin	1996	Ukraine	Krym, Karadagh
MM11438	Elachista OTU18	BOLD:AAB2133	632	KJ130880			Yu Budashkin	1996	Ukraine	Krym, Karadagh
MM15301	Elachista OTU14	BOLD:AAB2137	658	JF847598			J. Tabell	12-Apr-2010	Spain	Sitges
MM15302	Elachista OTU14	BOLD:AAB2137	658	JF847599			J. Tabell	22-Apr-2010	Spain	Sitges
MM15303	Elachista OTU14	BOLD:AAB2137	658	JF847600			J. Tabell	08-Apr-2010	Spain	Sitges, Viladellops
MM15304	Elachista OTU14	BOLD:AAB2137	658	JF847601			J. Tabell	26-Apr-2010	Spain	Sitges
MM15318	Elachista OTU13	BOLD:AAB3258	658	JF847615			T. Nupponen	18-Nov-2009	Tunisia	Kasserine, Djebel Chambi
MM15319	Elachista OTU13	BOLD:AAB3258	658	JF847616			T. Nupponen	18-Nov-2009	Tunisia	Kasserine, Djebel Chambi
MM15320	Elachista OTU13	BOLD:AAB3258	658	JF847617			T. Nupponen	18-Nov-2009	Tunisia	Kasserine, Djebel Chambi
MM15321	Elachista OTU6	BOLD:AAP4312	658	JF847618			T. Nupponen	18-Nov-2009	Tunisia	Kasserine, Djebel Chambi
MM15322	Elachista OTU6	BOLD:AAP4312	658	JF847619			T. Nupponen	18-Nov-2009	Tunisia	Kasserine, Djebel Chambi
MM15323	Elachista OTU6	BOLD:AAP4312	658	JF847620			T. Nupponen	18-Nov-2009	Tunisia	Kasserine, Djebel Chambi
MM15324	Elachista OTU6	BOLD:AAP4312	658	JF847621			T. Nupponen	18-Nov-2009	Tunisia	Kasserine, Djebel Chambi
MM15325	Elachista OTU12	BOLD:AAB2139	656	JF847622			T. Nupponen, K. Nupponen	22-Apr-2009	Spain	Andalusia, Almeria, Almeria
MM15329	Elachista OTU12	BOLD:AAB2139	658	JF847626			T. Nupponen, K. Nupponen	22-Apr-2009	Spain	Andalusia, Almeria, Almeria
MM15335	Elachista OTU12	BOLD:AAB2139	658	JF847632			T. Nupponen, K. Nupponen	22-Apr-2009	Spain	Andalusia, Almeria, Almeria
MM15336	Elachista OTU12	BOLD:AAB2139	657	JF847633			T. Nupponen, K. Nupponen	22-Apr-2009	Spain	Andalusia, Almeria, Almeria,
MM15339	Elachista OTU12	BOLD:AAB2139	658	JF847636			T. Nupponen, K. Nupponen	22-Apr-2009	Spain	Almeria
MM15340	Elachista OTU12	BOLD:AAB2139	658	JF847637			T. Nupponen, K. Nupponen	22-Apr-2009	Spain	Andalusia, Almeria, Almeria
MM15477	Elachista OTU9	BOLD:AAP4191	658	JF847742			T. Nupponen	12-Jul-2010	Spain	Andalusia, Granada, Sierra Nevada
MM15478	Elachista OTU7	BOLD:AAB2141	658	JF847743			T. Nupponen	15-Jul-2010	Spain	Castilla-La Mancha, Cuenca, Fuentes
MM15483	Elachista OTU5	BOLD:AAP4193	658	JF847748			T. Nupponen	12-Jul-2010	Spain	Andalusia, Granada, Sierra Nevada
MM15484	Elachista OTU17	BOLD:AAP4194	658	JF847749			T. Nupponen	11-Jul-2010	Spain	Andalusia, Granada, Sierra Nevada
MM16250	Elachista OTU5	BOLD:AAP4193	658	JF847451	M		J. Tabell	08-Jun-2010	Spain	Andalusia, Granada, Sierra Nevada, Puerto de la Ragua
MM16251	Elachista OTU5	BOLD:AAP4193	658	JF847452	M		J. Tabell	08-Jun-2010	Spain	Andalusia, Granada, Sierra Nevada, Puerto de la Ragua
MM16252	Elachista OTU5	BOLD:AAP4193	658	JF847453	M		J. Tabell	07-Jun-2010	Spain	Andalusia, Granada, Sierra Nevada, Puerto de la Ragua
MM16254	Elachista OTU14	BOLD:AAB2137	658	JF847454	M		J. Tabell	14-Jun-2010	Spain	Catalonia, Barcelona, near Barcelona
MM16255	Elachista OTU12	BOLD:AAB2139	658	JF847455	M		J. Tabell	11-Jun-2010	Spain	Andalusia, Granada, Cullar 5, 5 km N
MM16258	Elachista OTU4	BOLD:AAP4902	658	JF847457	M		K. Nupponen	28-Jun-2003	Russia	Orenburg, Orenburg, Donskoje vill. 6 km W
MM16259	Elachista OTU4	BOLD:AAP4902	658	JF847458	M		K. Nupponen	28-Jun-2003	Russia	Orenburg, Orenburg, Donskoje vill. 6 km W
MM16379	Elachista OTU14	BOLD:AAB2137	658	KJ130894	M		J. Tabell		Spain	Catalonia, Barcelona
MM16738	Elachista OTU13	BOLD:AAB3258	658	JF847564	F		P. Huemer	02-Sep-2005	Spain	Comunidad Valenciana, Alicante, Sierra de Crevillente, 5 km NE Albatera, 450 m

MM16739	Elachista OTU10	BOLD:AAI6215	658	JF847565	F		P. Huemer	02-Sep-2005	Spain	Comunidad Valenciana, Alicante, Sierra de Crevillente, 5 km NE Albatera, 450 m
MM16740	Elachista OTU10	BOLD:AAI6215	658	JF847566	M		P. Huemer	02-Sep-2005	Spain	Comunidad Valenciana, Alicante, Sierra de Crevillente, 5 km NE Albatera, 450 m
MM16741	Elachista OTU10	BOLD:AAI6215	658	JF847567	M		P. Huemer	02-Sep-2005	Spain	Comunidad Valenciana, Alicante, Sierra de Crevillente, 5 km NE Albatera, 450 m
MM16745	Elachista OTU15	BOLD:AAB3257	658	JF847571	M		P. Huemer	07-Sep-2005	Spain	Comunidad Valenciana, Valencia, El Saler, Albufera, 5 m
MM16765	Elachista OTU15	BOLD:AAB3257	658	KJ130875	M		Ch. Wieser	period 8.-9.8.2005	France	Narbonne, Salins de Saint Lucia
MM16766	Elachista OTU15	BOLD:AAB3257	658	KJ130936	M		Ch. Wieser	period 8.-9.9.2005	Spain	Comunidad Valenciana, Valencia, Albufera
MM16767	Elachista OTU15	BOLD:AAB3257	658	KJ130903	M		Ch. Wieser	period 8.-9.9.2005	Spain	Comunidad Valenciana, Alicante, Albufera
MM16771	Elachista OTU12	BOLD:AAB2139	658	KJ130904	M		Ch. Wieser	period 5.-6.9.2005	Spain	Comunidad Valenciana, Alicante, Santa Pola, Playa del Pinet, 5 m
MM16798	Elachista OTU13	BOLD:AAB3258	658	JF853401	M		Ch. Wieser	03-Sep-2005	Spain	Comunidad Valenciana, Alicante, Siera de Crevillente nr Albatera
MM16799	Elachista OTU15	BOLD:AAB3257	658	JF853402	M		Ch. Wieser	31-Aug-2005	France	Narbonne, Salins de Saint Lucia, 1 m
MM16801	Elachista OTU7	BOLD:AAB2141	656	JF853403	M		P. Huemer	31-Aug-2005	Spain	Castellon, Penyagolosa N-Hang Banyadera, 1500 m
MM16833	Elachista multipunctella		164		M	holotype	F. Kasy	01-May-1975	Austria	Austria inf. or., Hundsheimer Berg, Porta hungarica
MM16834	Elachista skulei		164		M	holotype	B. Skule, S. Langemark	28.-29.6.1982	Greece	Lakonia, Mt. Taygetos
MM16835	Elachista occidentella		164		M	holotype	O. Karsholt	08-Aug-1986	Portugal	Ext. 3 km E Capo da Roca
MM16837	Elachista vanderwolfi		56		M	holotype	H. W. v.d. Wolf	25.-27.7.1986	France	Drome, la Penneze-s-l-Ouveze
MM16838	Elachista minusculella		56		M	holotype	M. Glaser, W. Glaser	06-Jun-1965	Turkey	Istanbul, 50 km E. Istanbul
MM16839	Elachista blancella		164		M	holotype	M. Fibiger	24-Jul-1986	Turkey	Prov. Ankara, 20 km NW Kizilcan
MM16840	Elachista casascoensis		164		M	holotype	G. Baldizzone	07-Aug-1984	Italy	Piedmont, Val Curone (Pr. AL) Poggio de Casasco
MM16842	Elachista bazaella		94		M	holotype	G. Baldizzone, P. Triberti	25-Jul-1983	Spain	Andalusia, Sierra Nevada, Cam. De Veleta
MM16843	Elachista veletaella		56		M	holotype	E. Traugott-Olsen	24-Jul-1983	Spain	Andalusia, Sierra Nevada, Cam. De Veleta
MM16844	Elachista varensis		94		M	holotype	O. Karsholt, N. P. Kristensen	08-Apr-1988	France	Var, 20 km S. St. Tropez, Cap Cartaya, Plage de l`Escalet
MM16846	Elachista anitella		164		M	holotype	E. Traugott-Olsen	19-Aug-1984	Spain	Andalusia, Sierra Nevada, Cam. De Veleta
MM16847	Elachista senecai		93		M	holotype	U. Seneca	13-May-1983	Libya	Gharian, Wadi El Hira
MM16848	Elachista toveella		164		M	holotype	E. Traugott-Olsen		Spain	Andalusia, Sierra Nevada, Cam. De Veleta
MM16849	Elachista wadielhiraensis		164		M	holotype	U. Seneca	13-May-1983	Libya	Gharian, Wadi El Hira
MM16850	Elachista michelseni		56		M	holotype	Exp. ZMUC	14.-16.3.1986	Tunisia	Nefta
MM16851	Elachista bengtssoni		325	KJ130943	M	holotype	B. A Bengtsson	28-Jun-1989	Spain	Comunidad Valenciana, Alicante, Torrevieja, Lago Jardin
MM16852	Elachista rissaniensis		164		M	holotype	Exp. ZMUC	13.-14.4.1986	Morocco	Erfoud Rissani area
MM16853	Elachista berndtiella		164		M	holotype	E. Traugott-Olsen		Spain	Andalusia, Sierra Nevada, Cam. De Veleta
MM16854	Elachista olemartini		94		M	holotype	Exp. ZMUC	14.-16.3.1986	Tunisia	Nefta
MM16855	Elachista hispanica		94		M	holotype	E. Arenberger	03-Jul-1967	Spain	Catalonia, Collado de Falset
MM16857	Elachista povolnyi		56		M	holotype	F. Kasy	01-May-1976	Austria	Austria inf. Glaslauterriegel, S. Gumpoldskirchen
MM16858	Elachista intrigella		164		M	holotype	F. Kasy	27-Jul-1978	Austria	Austria inf. or., Hundsheimer Berg, Porta hungarica
MM16860	Elachista nielspederi		164		M	holotype	F. Kasy	01-Aug-1970	Austria	Austria inf. or., Hundsheimer Berg, Porta hungarica
MM16861	Elachista karsholti		94		M	holotype	F. Kasy	22-May-1971	Austria	Austria inf.Glaslauterriegel, Gumpoldskirchen
MM16863	Elachista imbi		164		M	holotype	F. Kasy	23-Jul-1977	Austria	Austria inf. or., Hundsheimer Berg, Porta hungarica
MM16866	Elachista oukaimedenensis		94		M	holotype	F. Kasy	10-Jul-1975	Morocco	Marrakech-Tensift-El Haouz Region, Al Haouz Province, High Atlas, 4 km E. Oukameden
MM16867	Elachista glaseri		94		M	holotype	M. Glaser, W. Glaser	1.-2.6.1973	Spain	Murcia, Alhama de Murcia
MM16868	Elachista moroccoensis (OTU19)	BOLD:ACD0666	658	KJ130908	M	holotype	Exp. ZMUC	12-Apr-1989	Morocco	Tinerhir region, - m
MM16869	Elachista baldizzonella		164		M	holotype	E. Traugott-Olsen		Spain	Andalusia, Sierra Nevada, Cam. De Veleta
MM16870	Elachista louiseae		56		M	holotype	G. Baldizzone, P. Triberti	25-Jul-1983	Spain	Andalusia, Sierra Nevada, Cam. De Veleta
MM16871	Elachista rikkeae		94		M	holotype	G. Baldizzone, E. Traugott-Olsen	17-Jul-1987	Spain	Andalusia, Granada, Cam.. Baza-Benamaurel, 15 km de Baza
MM16872	Elachista tribertiella		90		M	holotype	E. Traugott-Olsen	24-Jul-1983	Spain	Andalusia, Sierra Nevada, Cam. De Veleta
MM16873	Elachista gerdmaritella		94		M	holotype	E. Traugott-Olsen	30-Oct-1982	Spain	Andalusia, Malaga, Camino de Ojen
MM16988	Elachista OTU16	BOLD:AAV1042	658	KJ130935	M		P. Huemer	09-Aug-2010	Slovenia	Julijske Alpe, Veliki Mangart
MM16989	Elachista OTU16	BOLD:AAV1042	658	KJ130945	M		P. Huemer	09-Aug-2011	Slovenia	Julijske Alpe, Veliki Mangart
MM16991	Elachista OTU2	BOLD:AAQ0950	658	KJ130890	M		P. Buchner	17-Apr-2004	Austria	3 km SSW Katzeldorf
MM16992	Elachista OTU2		307	KJ130931	M		P. Buchner	17-Apr-2004	Austria	3 km SSW Katzeldorf
MM16993	Elachista OTU2	BOLD:AAQ0950	658	KJ130937	M		P. Buchner	03-Jul-2006	Austria	Eichkogel S Moedling
MM16994	Elachista OTU2	BOLD:AAQ0950	658	KJ130917	M		P. Buchner	15-Sep-2006	Austria	Eichkogel S Moedling
MM16995	Elachista OTU8	BOLD:AAB2134	658	KJ130939	F		P. Buchner	19-May-2006	Austria	Wr. Neustadt West
MM16996	Elachista OTU2	BOLD:AAQ0950	658	KJ130940	M		P. Buchner	14-Jul-2006	Austria	Eichkogel S Moedling
MM16997	Elachista OTU2	BOLD:AAQ0950	658	KJ130884	M		P. Buchner	24-Aug-2007	Austria	Bisamberg
MM16998	Elachista OTU2	BOLD:AAQ0950	634	KJ130918	M		P. Buchner	14-Jul-2006	Austria	Eichkogel S Moedling
MM18562	Elachista OTU2	BOLD:AAQ0950	658	KJ130895			Ingvar Svensson	01-Jun-1994	Sweden	Sm, Hoegsby
MM19857	Elachista OTU8	BOLD:AAB2134	621	JN267135			N. Savenkov	08-Jul-2000	Latvia	Rig., Garkalne
MM19979	Elachista OTU11		407	KJ130876			Zdenko Tokar		Croatia	Gospic
MM19992	Elachista OTU1	BOLD:AAI6216	407	KJ130883			Zdenko Tokar		Spain	Comunidad Valenciana, Alicante, Albatero env.
MM19998	Elachista OTU12	BOLD:AAB2139	658	KJ130878			Zdenko Tokar	20.-21.4.2009	Spain	Andalusia, Almeria, Tabernas env., Aghuilla Salada
MM19999	Elachista OTU12	BOLD:AAB2139	658	KJ130929			Zdenko Tokar	20.-21.4.2009	Spain	Andalusia, Almeria, Tabernas env., Aghuilla Salada
MM20049	Elachista OTU8	BOLD:AAB2134	564	JN267128	M		J. Heckford	01-Nov-2008	United Kingdom	Cornwall, Downas valley
MM20174	Elachista OTU13	BOLD:AAB3258	658	KJ130916	M		J. Tabell	12-Jun-2010	Spain	Comunidad Valenciana, Alicante, San Miguel de Salinas 3 km SW
MM20223	Elachista OTU8	BOLD:AAB2134	658	KJ130922	M		N. Savenkov	29-Jul-2000	Latvia	Riga, Garkalne
MM20504	Elachista OTU12	BOLD:AAB2139	407	KJ130928			Zdenko Tokar		Spain	Comunidad Valenciana, Alicante, Santa Pola env.
MM20507	Elachista OTU13	BOLD:AAB3258	407	KJ130888			Zdenko Tokar		Spain	Comunidad Valenciana, Alicante, Albatera env.
MM20510	Elachista OTU10	BOLD:AAI6215	630	KJ130907			Zdenko Tokar	9.-10.7.2010	Spain	Andalusia, Granada, Diezma
MM20519	Elachista OTU13	BOLD:AAB3258	407	KJ130925			Zdenko Tokar		Spain	Andalusia, Almeria, Tabernas env., Aghuilla Salada
MM20822	Elachista OTU16	BOLD:AAV1042	658	KJ130933	M		P. Huemer	09-Aug-2010	Slovenia	Julijske Alpe, Veliki Mangart
MM20824	Elachista OTU16	BOLD:AAV1042	658	KJ130905	M		P. Huemer	09-Aug-2010	Slovenia	Julijske Alpe, Veliki Mangart
MM20825	Elachista OTU16	BOLD:AAV1042	658	KJ130893	M		P. Huemer	09-Aug-2010	Slovenia	Julijske Alpe, Veliki Mangart
MM20826	Elachista OTU16	BOLD:AAV1042	658	KJ130889	M		P. Huemer	09-Aug-2010	Slovenia	Julijske Alpe, Veliki Mangart
MM20827	Elachista OTU16	BOLD:AAV1042	658	KJ130900	M		P. Huemer	09-Aug-2010	Slovenia	Julijske Alpe, Veliki Mangart
MM20828	Elachista OTU2	BOLD:AAQ0950	658	KJ130941	M		P. Huemer	01-Aug-2010	Italy	Prov. Cuneo, Valdieri
MM20829	Elachista OTU16	BOLD:AAV1042	658	KJ130927	M		P. Huemer	13-Jun-2007	Italy	Prov. Udine, Valle di Uccea, 3 km N Uccea
MM20840	Elachista OTU16	BOLD:AAV1042	658	KJ130879	M		P. Huemer	09-Aug-2010	Slovenia	Julijske Alpe, Veliki Mangart
MM20874	Elachista OTU12	BOLD:AAB2139	658	KJ130892	M		HW vd. Wolf	12-Apr-1997	Spain	Andalusia, Almeria, Mini-Hollywood, 6 km SW Tabernas
MM20875	Elachista OTU13	BOLD:AAB3258	658	KJ130896	M		HW vd. Wolf	12-Apr-1997	Spain	Andalusia, Almeria, Mini-Hollywood, 6 km SW Tabernas
MM21349	Elachista OTU5	BOLD:AAP4193	658	KJ130923	M		Z. Tokar	27-May-2001	Slovakia	Slov.-krasZadiel
MM21356	Elachista OTU8	BOLD:AAB2134	657	KJ130898	M		L. Srnka	15-Aug-2003	Croatia	South Velebit
MM21379	Elachista OTU8	BOLD:AAB2134	658	KJ130944	M		Z. Tokar	26-May-2004	Hungary	Csakbereny
MM21402	Elachista OTU3	BOLD:ABW1969	658	KJ130901	M		Z. Tokar	27-Jun-2003	Croatia	Tribunj
MM21403	Elachista OTU2	BOLD:AAQ0950	611	KJ130906	M		I. Richter	14-May-1996	Slovakia	Bojnice
MM21404	Elachista OTU2	BOLD:AAQ0950	652	KJ130919	M		Z. Tokar	27-Jun-2001	Slovakia	Slov. Kras-Vidova
MM21405	Elachista OTU2	BOLD:AAQ0950	658	KJ130915	M		Z. Tokar	24-Aug-2002	Slovakia	Kasvar
MM21406	Elachista OTU2	BOLD:AAQ0950	658	KJ130930	M		Z. Tokar	07-Jul-2001	Slovakia	Slov.kras-Turna
MM21418	Elachista OTU2	BOLD:AAQ0950	658	KJ130909	M		Z. Tokar	07-Aug-2001	Slovakia	Plesivecka plan
MM21419	Elachista OTU2	BOLD:AAQ0950	410	KJ130920	M		Z. Tokar	04-May-2002	Slovakia	Slov. Kras-Hrhov
MM21432	Elachista OTU1	BOLD:AAI6216	658	KJ130902	M		J. Tabell	15-May-2009	Spain	10 km NNW Albatera
MM21433	Elachista OTU1	BOLD:AAI6216	658	KJ130926	M		J. Tabell	23-Apr-2009	Spain	3 km SW S. Miguel de Salinas
MM21436	Elachista OTU18	BOLD:ACE9135	658	KJ130934	M		A. Lastuvka	21-Jul-1993	France	Luberon mts., La Motte d`Aigues
